# PRIESSTESS: interpretable, high-performing models of the sequence and structure preferences of RNA-binding proteins

**DOI:** 10.1093/nar/gkac694

**Published:** 2022-08-26

**Authors:** Kaitlin U Laverty, Arttu Jolma, Sara E Pour, Hong Zheng, Debashish Ray, Quaid Morris, Timothy R Hughes

**Affiliations:** Department of Molecular Genetics, University of Toronto, Toronto, Canada; Department of Molecular Genetics, University of Toronto, Toronto, Canada; Donnelly Centre, University of Toronto, Toronto, Canada; Department of Molecular Genetics, University of Toronto, Toronto, Canada; Donnelly Centre, University of Toronto, Toronto, Canada; Donnelly Centre, University of Toronto, Toronto, Canada; Department of Molecular Genetics, University of Toronto, Toronto, Canada; Computational and Systems Biology, Memorial Sloan Kettering Cancer Center, New York, USA; Department of Molecular Genetics, University of Toronto, Toronto, Canada; Donnelly Centre, University of Toronto, Toronto, Canada

## Abstract

Modelling both primary sequence and secondary structure preferences for RNA binding proteins (RBPs) remains an ongoing challenge. Current models use varied RNA structure representations and can be difficult to interpret and evaluate. To address these issues, we present a universal RNA motif-finding/scanning strategy, termed PRIESSTESS (Predictive RBP-RNA InterpretablE Sequence-Structure moTif regrESSion), that can be applied to diverse RNA binding datasets. PRIESSTESS identifies dozens of enriched RNA sequence and/or structure motifs that are subsequently reduced to a set of core motifs by logistic regression with LASSO regularization. Importantly, these core motifs are easily visualized and interpreted, and provide a measure of RBP secondary structure specificity. We used PRIESSTESS to interrogate new HTR-SELEX data for 23 RBPs with diverse RNA binding modes and captured known primary sequence and secondary structure preferences for each. Moreover, when applying PRIESSTESS to 144 RBPs across 202 RNA binding datasets, 75% showed an RNA secondary structure preference but only 10% had a preference besides unpaired bases, suggesting that most RBPs simply recognize the accessibility of primary sequences.

## INTRODUCTION

Eukaryotic genomes encode hundreds to thousands of RNA-binding proteins (RBPs) which play diverse roles in RNA metabolism and gene regulation (1). A hallmark of RBPs is their ability to recognize specific RNA sequences and structures, and characterizing these RNA binding specificities is imperative for understanding RBP function (2). RNA structure has a significant and complex role in RNA–protein binding, ranging from the recognition of specific RNA stem and loop structures by RBPs (e.g. SLBP and LARP6 (3,4)), to the preferential binding of unpaired bases by sequence-specific RBPs (5). Many RBPs contain multiple RNA binding domains (RBDs), thus introducing additional complexities such as RBD binding to multiple shorter motifs separated by variable spacing (6–8). The first large-scale survey of *in vitro* secondary structure preferences found that 28% (54 of 193) of RBPs showed a statistically significant preference for RNA secondary structure (or its absence) (9). A subsequent re-analysis of these data (10) found that addition of RNA structural information improved identification of *in vitro* binding sites to some extent for all 205 studied RBPs. These analyses used binding data from mostly unstructured RNA probes, however. An independent analysis of 78 RBPs reported that 35% bound to a bipartite motif with a gap of variable length, 50% bound to more than one distinct motif, and found that RBP binding is often affected by the RNA sequence and structural context surrounding the binding motif (7). Another study of 86 RBPs reported that 29% bound to a bipartite motif, 48% bound to more than one motif, and 30% displayed a preference towards binding a motif in a hairpin loop structure (11). Although these studies employed different RNA binding assays and analysis methods, they collectively suggest that a substantial proportion of RBPs recognize RNA secondary structure and that bipartite RNA binding motifs and the ability to bind multiple motifs are surprisingly common. This outcome underscores the need for new RNA binding models that can capture diverse modes of RBP binding (12).

In living cells, binding sites of an RBP are commonly identified by immunoprecipitating RBP-RNA complexes, typically following UV cross-linking and querying the bound RNA by high-throughput sequencing (e.g. CLIP-seq and related approaches) (13). While conceptually straightforward, downstream processing of these data to identify *bona fide* binding sites remains a topic of active research (14). Additionally, these studies can be confounded by RNA binding sites that are influenced by cellular factors, and limited to sequences present in cellular RNA (12,15). In contrast, current *in vitro* RNA selection systems capture intrinsic sequence and structure binding specificities via direct RBP binding to complex pools of RNA probes followed by high-throughput sequencing of bound RNA. Four such methods have been described, which differ in technical details: RNA Bind-n-Seq (RBNS) (16), SEQRS (17), RNAcompete-S (18) and HTR-SELEX (11). While RBNS and RNAcompete-S are single-step competitive binding reactions, HTR-SELEX and SEQRS use multiple rounds of selection to enrich for high affinity binding targets. Taken together these experiments have been performed on 144 distinct RBPs, providing a large resource for investigation of RNA sequence and structure specificity.

RBP sequence specificities are often modelled as Position Frequency Matrices (PFMs), commonly called ‘motifs’ (19). The same approaches used for the derivation of (and scanning with) primary sequence motifs for DNA binding proteins can be applied to RBPs. The treatment of secondary structure information, however, is less standardized, and the representation of multiple motifs and bipartite motifs with variable gaps even less so. RNA base identity can be extended to include RNA secondary structure information, resulting in structure-augmented *k*-mers or PFMs (18,20). Derivation and representation of RNA secondary structure varies widely in RBP structure specificity modelling: RNA folding techniques differ between methods, and per-base RNA structural contexts are described using diverse ‘alphabets’ (e.g. 2-letter paired vs. unpaired alphabet (2), 5-letter alphabet incorporating stem and loop information (20), more complex schemes like BEAR (21)), with or without retention of specific base pairings. Virtually no two methods modelling RBP structure specificity use the same combination of structure derivation and representation. A host of deep learning methods have also been described, which are designed to incorporate secondary structure (22–31). These approaches generally require post hoc dissection, however, and to our knowledge there has been no systematic comparison among methods.

The diversity of sequence-structure specificity modelling is illustrated in the following four methods: RCK (32), GraphProt (33), pysster (22) and RNAcompete-S (18). All of these methods aim to identify the RNA sequence and structural specificity of RBPs from RNA binding data (i.e. a set of bound RNA probes) using distinct algorithms, generating models that can be used for scanning and scoring RNA sequences. RCK infers a sequence- and structure-based *k*-mer model from a set of RNA sequences and associated RNA structure context probabilities, which are derived from the ensemble of possible structures for each RNA probe. GraphProt determines three possible structures for each input RNA sequence and encodes them as graphs to preserve base-pairing information along with annotating bases with specific structural contexts. These graphs are used to define the kernel for a support vector machine model that captures both sequence and structural specificity. Pysster uses the minimum free energy structure of an RNA probe along with its nucleotide sequence to encode each RNA as a string that combines sequence and structure information. These strings are then used to train a convolutional neural network to identify sequence-structure motifs. RNAcompete-S determines RNA probe centroid structure and, similarly to pysster, encodes each probe with an alphabet that defines both primary sequence and secondary structure per base. These annotations are used as input for logistic regression to identify sequence-structure motif models, which can then be concatenated to create composite motifs. Across the four methods, three different sets of structural contexts, and therefore three different structure alphabets, are used to annotate the RNA secondary structure. The alphabets are composed of 4, 6, 4 and 7 structural contexts, respectively. Furthermore, each method was evaluated on a different type of RNA binding data with different biases and properties, and consequently it is difficult to compare their performance. Finally, other than pysster, each method produces only a single representative binding motif to describe the RNA binding specificity; although RCK and GraphProt theoretically should be able to model multiple specificities simultaneously, that information is not made available to the user.

Here we present PRIESSTESS (**P**redictive **R**BP-RNA **I**nterpretabl**E S**equence-**S**tructure mo**T**if regr**ESS**ion), a method that is conceptually related to the computational component of RNAcompete-S, but automates and significantly extends most of the process. PRIESSTESS captures sequence and structure specificity from *in vitro* RBP–RNA binding data and was designed specifically to address the diversity of RBP binding modes: the variation in RNA secondary structure recognition and the potential for multiple and/or bipartite motifs. Importantly, the method is designed to be modular, transparent, and flexible. It produces models that are readily interpretable, with all motifs made available to the user, provides an inherent measure of RBP structure specificity, and facilitates scanning for motifs which is less straightforward with other methods. We also describe a new data assembly, including new HTR-SELEX data for 23 proteins, which we use to show that PRIESSTESS performs comparably to other models in general while providing greater interpretability of RBP specificity. Finally, we use PRIESSTESS to gauge the structural specificity of 144 RBPs with *in vitro* RBP-RNA binding data and find that the dominant RNA structural attribute across all models is a propensity to bind unpaired bases.

## METHODS

### HTR-SELEX

We ran new HTR-SELEX experiments as described in (11) with the following modifications. (i) We updated the selection ligand (N40 with flanking index and Illumina primers) for compatibility with the current Illumina sequencing platforms (see Supplementary Table S1 for primer sequences). (ii) We employed GST fusion proteins instead of a thioredoxin-His6-tag. Sequences for the 23 constructs are indicated in Supplementary Table S2. We produced proteins in *Escherichia coli* as described in (34); glutathione coated magnetic affinity beads used for immobilizing RBP-RNA complexes were purchased from Sigma (Sigma Aldrich, cat. nr. G0924). (iii) The washing protocol utilized a BioMex FX liquid handler instead of a Biotek405 plate washer. Data sets are on the European Nucleotide Archive (accession: PRJEB47428).

### RBP binding data

We downloaded RNAcompete-S data (18) from NCBI (Accession: PRJNA298655). For each of the 78 RBPs with RBNS data (7), we downloaded the binding data from the best concentration as defined in Dominguez *et al.* from ENCODE (35), along with the associated input library to serve as a negative set. We downloaded the first and fourth cycle (used as the negative and positive sets, respectively) of HTR-SELEX experiments from Jolma *et al.* (11) from the European Nucleotide Archive (Accession: PRJEB25907). First cycles were used, rather than the input pool (i.e. zero cycle), because the input pools can be shared across multiple experiments, resulting in common signals, which we attribute to some aspect of the pool, possibly because it is sequenced at a different time. The starting RNA pool was used as the negative set for a small number of experiments that lacked sequencing data for the first cycle, or that had <100 000 RNA probes sequenced in the first cycle. We only analyzed the 95 experiments (86 RBPs) for which Jolma *et al.* obtained a Position Weight Matrix (PWM). A further 23 HTR-SELEX experiments were also performed for this paper (as described above), for which the first and fourth cycles were used as the negative and positive set, respectively. Details for all four datasets can be in found in Supplementary Tables S3–S6.

We discarded RNA probes containing ambiguous, uncalled bases from all datasets, and removed probes containing part of the flanking primer sequence in RBNS data. Negative and positive sets were subsampled to 1 million sequences each, to avoid long compute times when identifying enriched motifs. For experiments with fewer than 1 million negative or positive probes, we subsampled the larger set to match the number of probes in the smaller set. The positive and negative sets for each RBP experiment were split into 75% training data and 25% testing (held-out) data.

We obtained matching eCLIP (36) experimental data (for the same RBP or a close homologue) from ENCODE. Specifically, we downloaded the BED files with merged peaks from both replicates. We extended eCLIP peaks by 20 bases upstream, as the 5’ end of the peak corresponds to the cross-linking site. These data were supplemented with other published CLIP data for ELAVL1 (37), Mbnl1 (38), Nova2 (39) and RC3H1 (40). Peaks from ELAVL1 and Mbnl1 BED files were not extended in either direction as they encompassed the binding site. RC3H1 and Nova2 peaks were narrow (<9 bases on average), thus we extended all peaks from these datasets by 15 bases on both sides to capture the full binding site. We generated negative sets for all CLIP experiments by shifting the peak location 300 bases upstream (as in (10)). Details on *in vivo* datasets can be found in Supplementary Tables S7 and S8.

### Folding and annotating structures

To annotate probe structure, each was folded using RNAfold with the -p option (41) (centroid structure) at the appropriate temperature for each experiment (4 or 21°C for RBNS experiments, 37°C for all other experiments). Using the output dot-bracket structure, each probe was labeled with a structural alphabet describing seven different structural contexts as in Cook *et al.*: E—unpaired/external, B—bulge loop, L—left paired base, R—right paired base, T—internal loop, M—multi-loop, H—hairpin loop (18). In this alphabet, a bulge loop is defined as an internal loop with unpaired bases on only one side of the stem. The probe sequence (4-letter alphabet) and 7-letter structure annotation were then combined to generate a 28-letter sequence-structure annotation including each possible combination of nucleotide and structure context. Similarly, the dot-bracket structure was also annotated using a 4-letter structure alphabet (P—paired base, L—hairpin loop, U—unpaired/external, M—multi-loop, internal loop or bulge loop) and a 2-letter structure alphabet (U—unpaired base, P—paired base). These annotations were combined with the sequence alphabet to generate a 16-letter and an 8-letter sequence-structure annotation, respectively. Thus, every probe has seven distinct annotations that are used downstream: sequence-only, structure-only (2-, 4-, or 7-letter alphabet), sequence-structure (8-, 16- or 28-letter alphabet).

There are several other options for calculation and representation of RNA structure (in addition to the centroid, as employed above), including the minimum free energy (MFE) structure and retention of one or more suboptimal structures. To determine if these representations would better model probe structure, we identified the representation that contained the largest enrichment of the known sequence-structure binding motif for hairpin binding and complex structure binding RBPs. We folded a subset of probes from the positive and negative sets of each experiment to determine the MFE structure, the centroid structure, and 1, 2 or 3 randomly sampled suboptimal structures per probe (RNAsubopt) with Vienna tools (41). We counted the occurrence of regular expressions representing the literature-described sequence-structure preference of each RBP (Supplementary Table S9). The centroid structure had the highest fold change in the positive over negative sets and was used in all further analysis (Supplementary Figure S1).

HTR-SELEX and RBNS experiments incorporate constant flanking sequences in the randomized RNA probes that are present during the binding step. For these datasets, the full-length probe including the flanks was folded and annotated; however, only the sequence and structure of the variable region of the probe was used for downstream analysis. Flanking sequences can be found in Supplementary Tables S3–S6. Similarly, 50 bases were added upstream and downstream of each CLIP peak (on top of any extensions described previously) for folding and were then removed prior to subsequent steps.

### Identification of enriched motifs

Using two-thirds of the training data we identified enriched PFMs with STREME (42). This was done seven times, using each of the seven probe annotations described above. We ran STREME with the options -pvt 0.01 -minw 4 -maxw 6 -alph <X>, with <X> representing the path to the file holding the alphabet definition for the probe annotation. The -pvt option limits motifs to those with a *P*-value of <0.01 and the -minw and -maxw options restrict motif size to between 4 and 6 bases in length, allowing the identification of shorter, partial motifs as well as motifs of standard size. For each of the seven sets of PFMs identified by STREME, we applied Bonferroni correction to the *P*-values and extracted up to 40 PFMs. We scanned these PFMs on the remaining one-third of the training data (the data not used for STREME), scoring the PFM at each base. For each PFM, we calculated the sum of the top four scores per probe. This was used as input to train the Logistic Regression (LR) and Random Forest (RF) models.

### Logistic regression and random forest models

We used the LogisticRegression function from scikit-learn (43,44) along with BayesSearchCV (45) from scikit-optimize (https://scikit-optimize.github.io) to determine the optimal L1 (i.e. LASSO) regularization strength using 80% of the scanned probes (80% of the data not used for STREME). BayesSearchCV was set to only optimize the L1 value. The initial Area Under the Receiver Operating Characteristic (AUROC) was then computed on a validation set consisting of the remaining 20% of the data.

To simplify the model, we reiterated the process, scaling the L1 regularization strength parameter by 1.25 at each iteration, until the AUROC dropped by 10% of the predictive power (predictive power is taken as *initialAUROC - 0.5*).

We used the RandomForestClassification function from scikit-learn along with BayesSearchCV from scikit-optimize to determine the optimal model (44). BayesSearchCV was set to optimize the number and depth of the decision trees in the model.

### Hidden Markov models

Using the PFMs from each of seven alphabets, we initialized and trained seven Hidden Markov Models (HMMs) (46) using the MultinomialHMM function from the hmmlearn package (https://hmmlearn.readthedocs.io/). States in the model include recursive ‘begin’ and ‘end’ states, a state for each position in the PFMs used, and recursive ‘gap’ states (Supplementary Figure S2). We set start probabilities such that each probe must start in the ‘begin’ state. We set transition probabilities to allow the ‘begin’ state and each state representing the final position in a PFM to transition directly to the states representing the first position in all PFMs. This allows two PFMs to occur back-to-back. States representing the first to the second last position in the PFMs are only able to transition to next position within the PFM, forcing a PFM to occur in its entirety. Recursive ‘gap’ states exist for each pair of PFMs in the model and allow modelling of gaps between two PFMs. The observations for the HMM are the letters in the alphabet from which we identified the PFMs. We set emission probabilities in PFM states to be equal to the probabilities in the PFM, and for all other states the probabilities for each observation were set to be 1/(alphabet length). Training occurred without updating the emission probabilities, leaving the information in the PFMs intact.

Due to runtime restraints, we initialized HMMs with a maximum of 10 PFMs and trained on a maximum of 10 000 RNA probes from the one-third of the positive set that was not used for STREME. If fewer than 20 000 probes were available for training, we used half. After we trained an HMM for each alphabet, we computed the log probability under the model for the remainder of the unused positive training set probes and a matching amount of unused negative training set probes. As input to a RF model, we used log probabilities for all seven HMMs. We used the RandomForestClassification function from scikit-learn along with BayesSearchCV from scikit-optimize to determine the optimal model. BayesSearchCV was set to optimize the number and depth of the decision trees in the model.

### RCK, GraphProt, BEESEM and pysster

We ran RCK (32) with default parameters and *k*-mer size set to 6. We also ran GraphProt (33) with default parameters, however, due to runtime, all RNAcompete-S and RBNS training sets were reduced to 375 000 probes from 750 000 (75% of the full set of 1 million). Similarly, we ran BEESEM (47) with default parameters, however, as BEESEM is designed for transcription factors, identified PWMs may be reverse complemented. Thus, we scored both the output PWM and its reverse complement against the held-out set and retained the PWM that produced a higher AUROC for all other testing.

Each pysster (22) model was trained with two convolutional layers and 16 filters of length 6. As pysster models can only be used for prediction on sequences of the same length as the training set sequences, a second set of CLIP testing data was generated by reducing each sequence to the same length as the RNA probes used to train the model, with the centre of this sequence at the CLIP peak. For accurate comparison, in Figure 3H, PRIESSTESS AUROCs were derived by applying the PRIESSTESS models to the same CLIP test sets as pysster. Furthermore, due to differing lengths of RNA probes in benchmarking dataset experiments, not all pysster models could be applied to all relevant *in vitro* datasets for testing, thus there are fewer comparisons in Figure 3I.

## RESULTS

### PRIESSTESS overview

**Figure 1.**
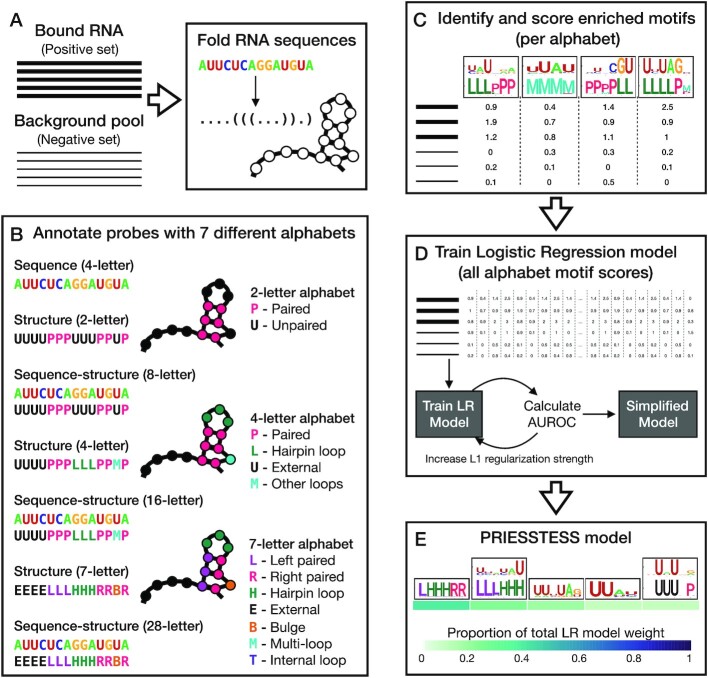
The PRIESSTESS method. (**A**) A set of RNA probes bound by an RBP of interest (positive set) and a set of probes from a background pool (negative set) are folded to determine the centroid structures using RNAfold (41). (**B**) Probes are annotated with seven different alphabets. Alphabets that include both sequence and structure information have a letter to represent each combination of secondary structure context and primary nucleotide. For example, in the sequence-structure alphabet using the 2-letter structure alphabet there are 8 letters. (**C**) Using each probe annotation individually, motifs (PFMs) enriched in the positive set over the negative set are identified with STREME (42) using two-thirds of the training data. The PFMs are then scored on the remaining third of the training data, specifically, the sum of the top four scores per PFM per probe is calculated. Depicted example is for the 16-letter alphabet. (**D**) Scores for PFMs from all 7 sets of probe annotations are combined and used as features for training a Logistic Regression (LR) model with L1 regularization. After optimizing the L1 regularization strength using Bayesian optimization, the AUROC is calculated on a separate validation set. To simplify the model, the L1 regularization strength parameter is scaled by 1.25 and retrained and tested on the validation set. This process repeats iteratively, until the AUROC drops by 10% of the predictive power (predictive power is taken as *initial AUROC- 0.5*). (**E**) The final simplified LR model represents the PFMs that most significantly contribute to the RBP binding specificity and are associated with weights (coefficients) that indicate their importance. Here weights are shown for each PFM as the proportion of the total model weight.

PRIESSTESS consists of two steps. The first step generates a large collection of enriched motifs encompassing both RNA sequence and structure. The second step produces an aggregate model, which combines the motif scores into a single value, and gauges the relative importance of each motif. Figure 1 shows a schematic of the method; the Methods section provides details on implementation. The location of code and datasets required to implement the system and reproduce this work are available on GitHub (see Availability).

As input, PRIESSTESS requires a set of RNA sequences bound by an RBP and a set of background RNA sequences. In the first step, two-thirds of the input training data are used to identify motifs (i.e. PFMs) enriched in the set of bound RNAs, relative to the background, using STREME (42), and using seven different alphabets describing RNA sequence, structure, or both. The three RNA structure alphabets used here range from simple accessibility to a 7-letter alphabet covering multiple loop types (Figure 1A–C). The scores of these motifs on the remaining RNA probes in the training set are used as features to train a logistic regression (LR) model, using Bayesian optimization to set the L1 (i.e. LASSO) regularization strength (44,45). To produce a robust and simplified model of the RNA sequence and structure specificity, L1 strength is iteratively increased until the AUROC of the model on an internal validation set retains only 90% of the predictive power of the initial model (Figure 1D); as we show below, this step significantly reduces the number of motifs in the model, with no loss of performance on independent data. The final PRIESSTESS model thus retains only the motifs which provide the greatest predictive power in binding site identification, each with a coefficient, or weight, describing the magnitude of the contribution to the model (Figure 1E).

This series of steps was designed to solve several problems in the representation of RNA binding sites and to simultaneously facilitate both sequence scanning and interpretation of the models, including dissection of the relative weight of individual motifs in binding to RNA. Traditional motifs are easily visualized, unlike collections of *k*-mers that must be interpreted post hoc. Furthermore, as multiple motifs can be retained, PRIESSTESS models can include components of bipartite motifs and/or multiple distinct motifs for an individual RBP. The LR step integrates the motifs into a scanning model that outputs a single score, and also provides a measure of the contribution of each motif (the model coefficient), in particular revealing the contribution of RNA structure relative to primary sequence alone. Unlike previous methods, PRIESSTESS manages variation in structural specificity across RBPs: in the annotation step, PRIESSTESS includes multiple annotations of RNA probes with differing degrees of structural representation, including no structural representation and no sequence representation. As L1 strength is increased in the LR step, unnecessary motifs are dropped from the model, thus, RNA structural information is modelled only when it is relevant. PRIESSTESS is intentionally modular and is assembled from established tools, making it amenable to modification.

### Benchmarking dataset

**Figure 2.**
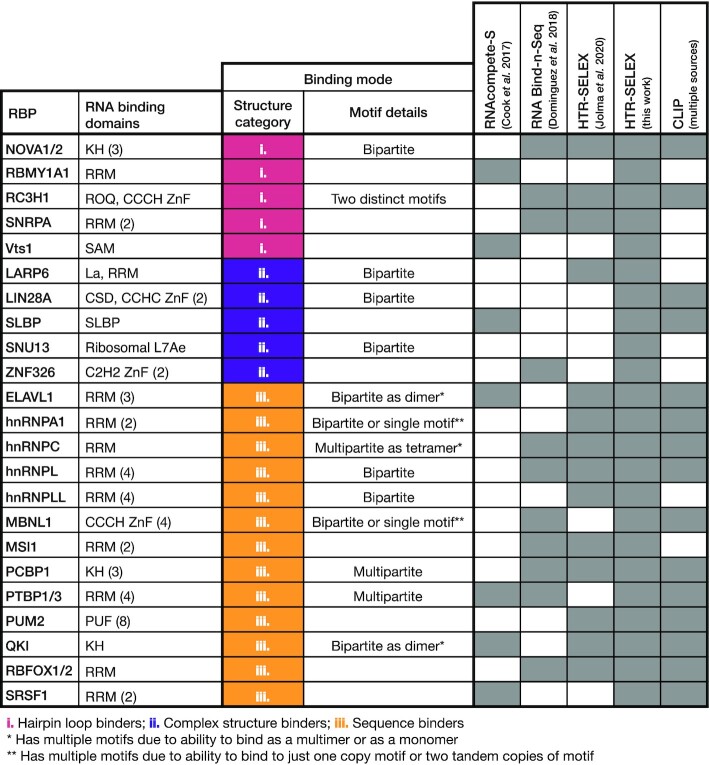
Benchmarking dataset. RBPs in the benchmarking dataset are listed along with their RNA Binding Domains (RBDs). Numbers in parentheses indicate the number of a given type of RBD in an RBP (e.g. KH (3) indicates three KH domains). The binding mode of each RBP as described in the literature is summarized in the third and fourth columns. RNA secondary structure binding type categories are as follows: i. ‘hairpin loop binders’ recognize a specific primary nucleotide sequence in a hairpin loop; ii. ‘complex structure binders’ recognize a specific primary sequence and secondary structure that is not a hairpin loop (e.g. double stranded RNA, internal loops); iii. ‘sequence binders’ have not previously been described as consistently and specifically binding to an RNA secondary structure beyond accessible sequence. The ability to bind to multiple and bi-/multi-partite motifs is also indicated. The availability of datasets from different assays is indicated by grey cells in the right-most columns. CLIP datasets are taken from multiple sources (36–39).

To assess PRIESSTESS we curated a collection of 23 RBPs with well-described binding specificities that span three categories: (i) RBPs that bind to a specific RNA sequence explicitly within a hairpin loop structure (e.g. SNRPA and Roquin/RC3H1 (48,49)), (ii) RBPs that bind to a specific sequence within another, often more complex, type of RNA structure (internal loop, double stranded RNA, etc.) (e.g. LARP6 and ZNF326 (4,7)), and (iii) well-characterized RBPs that primarily recognize unstructured primary nucleotide sequences (e.g. ELAVL1 and RBFOX1 (50,51)) (Figure 2). This collection also encompasses RBPs that bind to bipartite sites with variable spacing or to more than one distinct motif (e.g. HNRNPL and Roquin/RC3H1 (49,52)). RBPs were chosen based on the availability of previous *in vitro* binding data, for comparison (indicated in Figure 2) from RNAcompete-S (18), RBNS (7), and/or HTR-SELEX (11). Although no *in vitro* RNA selection data for LIN28A or SNU13 are available, both were added to the dataset to increase the number of complex structure binders. To complete and supplement the benchmarking data set, we also performed new HTR-SELEX experiments for all chosen RBPs (see Methods), resulting in a total of 55 experimental data sets. Among the 23 proteins in the benchmarking data set, 15 have CLIP data in the literature, spanning the three categories of RBPs, and encompassing proteins with bipartite binding (Figure 2, Supplementary Table S10).

Experiments were subsampled to a maximum of one million probes in each of the positive and negative datasets and split randomly for training (75% – 50% for motif identification; 20% for LR model training; 5% for LR model validation) and a held-out set for testing (25%). Negative sets were constructed from the input RNA pool for RBNS and RNAcompete-S experiments, and from the first selection cycle for HTR-SELEX experiments (see Methods for details). Despite the large proportion of held-out probes, results were reproducible with different training and testing splits (Supplementary Figure S3).

The running time of PRIESSTESS is largely dependent on the number of RNA probes used to train the model and the length and construction of the RNA probes, with runtime increasing linearly with dataset size and probe length (Supplementary Figure S4). For experiments in the benchmarking dataset with between 80 000 and 120 000 RNA probes in each of the positive and negative training sets, the median runtime is ∼4 h and 23 min on a single 2.4 GHz core.

### Comparison of PRIESSTESS to other methods

We compared the performance of PRIESSTESS to the published sequence-structure motif identification methods RCK (32), GraphProt (33) and pysster (22). We applied each of these methods to the training component of the benchmarking dataset and compared the performance of each method using the Area Under the Receiver Operating Characteristic (AUROC) curve on several different test sets (Supplementary Tables S11 and S12). AUROC quantifies the ability to distinguish ‘positive’ sequences bound by the RBP and ‘negative’ sequences drawn from the input pool or first selection cycle.

**Figure 3.**
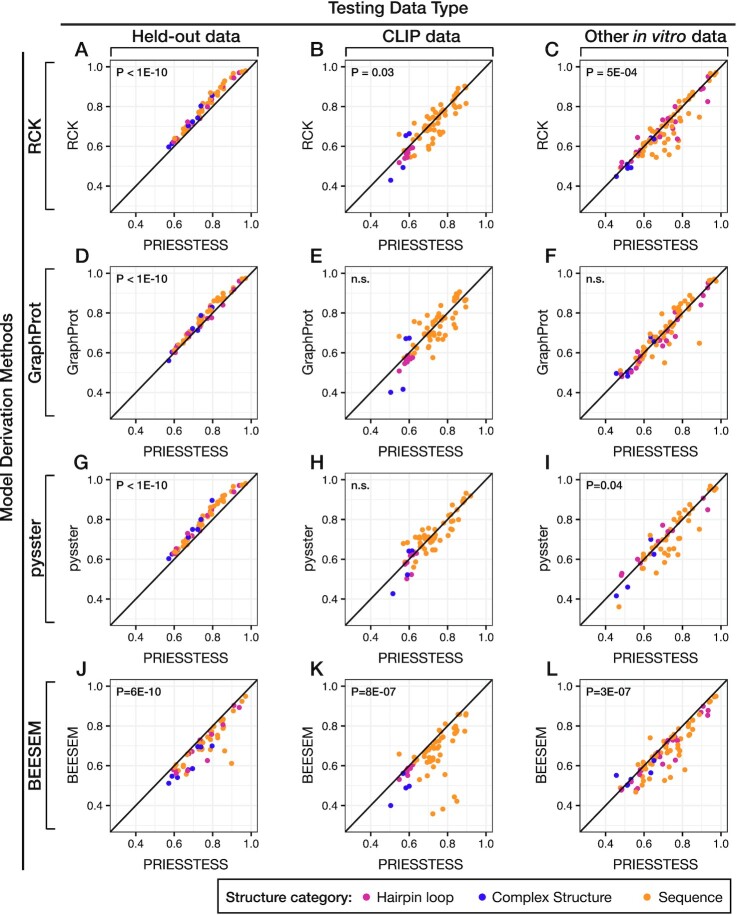
Comparison of PRIESSTESS performance to representative published alternatives. PRIESSTESS models trained on the benchmarking dataset are compared to RCK (32) (**A**–**C**), GraphProt (33) (**D–F**), pysster (22) (**G–I**) and BEESEM (**J–L**) (47) models trained using the same data. Plots show AUROC values of the trained models when applied to three different types of testing data: held-out data from the same experiment as the training data (A, D, G, J), matching CLIP data (B, E, H, K), and held-out data from other *in vitro* experiments (same RBP, different assay or different lab) (C, F, I, L). *P*-values resulting from two-sided paired *t*-tests are shown on each plot.

We first examined performance on the held-out test data sets. The methods are roughly comparable in predictive capacity over a wide range of AUROC values (Figure 3A, D, G), suggesting that model performance is limited primarily by the data itself. In these tests, RCK, GraphProt, and pysster models outperform PRIESSTESS (all *P* < 1E–10; paired t-test). At the task of discriminating between CLIP bound sequences and corresponding negatives, however, PRIESSTESS models trained on the *in vitro* benchmarking datasets outperform corresponding models generated by RCK (*P* = 0.03, paired *t*-test) and display no significant difference in performance compared to GraphProt and pysster models, each method outperforming the others on some RBPs (Figure 3B, E, H, Supplementary Tables S11 and S12). Moreover, when we tested across different *in vitro* datasets (e.g. training on HTR-SELEX and testing on RBNS held-out data (Figure 3C, F, I)), PRIESSTESS significantly outperforms RCK (*P* = 5E–04; paired *t*-test, effect size = 0.020) and pysster (*P* = 0.04, paired *t*-test, effect size = 0.016), and performs comparably to GraphProt. Thus, while no method displays a clearly superior performance in identifying *in vivo* binding sites, PRIESSTESS models tend to better represent the intrinsic *in vitro* binding specificity. It is possible that this difference is due to the fact that RCK, GraphProt, and pysster models retain considerably more information than PRIESSTESS (e.g. a full index of *k*-mer parameters, instead of PFMs) and are therefore capable of learning subtle biases (e.g., in base content) that are shared by sequences within a given experimental dataset, but are different between datasets. As noted above, the PRIESSTESS models used in these analyses were obtained by a model simplification process, and we speculated that more complex models may also learn experiment specific biases that are removed by simplification. The original (i.e. unsimplified) LR models, however, display no significant difference in scoring on CLIP datasets and independent *in vitro* datasets, relative to the reduced (i.e. simplified) models (Supplementary Figure S5), indicating that the simplification process does not impact the prediction performance on independent data.

We further compared PRIESSTESS models to motifs identified from the same training data using BEESEM (47). BEESEM was created to identify a single maximally-predictive sequence PWM from HT-SELEX data using one cycle as positive set and an earlier cycle as a negative set. PRIESSTESS models outperform BEESEM models on held-out data, CLIP data, and data from other *in vitro* experiments (all *P* ≤ 8E−07; paired *t*-test) (Figure 3J–L). Similarly, on all three testing datasets, the single best sequence motif identified by STREME is outperformed by PRIESSTESS models (Supplementary Figure S6A, D, G). This outcome suggests that the more complex PRIESSTESS models better capture the intrinsic binding specificity through the inclusion of multiple motifs for an RBP, structure information, or both.

### Dissection of PRIESSTESS

Taking advantage of its modularity, we next used the benchmarking dataset of 23 RBPs across 55 experiments to interrogate how several possible modifications impact PRIESSTESS’s performance. We first tested whether the model could be improved by accounting for spacing and orientation preferences of multiple distinct motifs on the same RNA, which would be anticipated for multi-RBD proteins or proteins that homodimerize, and has been previously described for some of the RBPs in our dataset (4,53). To allow for the possibility of variable spacing, we trained a separate Hidden Markov Model (HMM) (46) to learn patterns and relationships between the motifs within each of the seven sequence/structure vocabularies, and then combined the seven HMM scores using random forests (44), to provide a single score for each sequence. This process did reveal known spacing and orientation preferences for several proteins in our dataset (Supplementary Figure S6J–N), and therefore may be valuable for further study of the RNA binding activities. It had no significant impact on scoring data from CLIP experiments or from other *in vitro* experiments (Supplementary Figure S6E, H), however, and was therefore not included in the default pipeline. We cannot rule out that CLIP datasets have insufficient power to detect these constraints, due to a small number of binding sites and the impact of indirect binding. Likewise, the *in vitro* experiments utilize random sequence DNA in which more complicated sequences tend to be more rare, and therefore make a smaller contribution to scoring. Nonetheless, it is also possible that the HMM step removes as much information as it adds.

We further tested whether any improvement was obtained by employing Random Forests (RF) in the main PRIESSTESS framework, which would have a better capacity than LR to learn non-additive relationships among motifs, such as ANDs and XORs. Supplementary Figure S6C, F, I shows that, though RF models significantly outperform LR models on held-out data (*P* < 1E−10; paired *t*-test), LR models significantly outperform RF models on CLIP data (*P* = 9E−04; paired *t*-test) and other *in vitro* data (P = 2E−03; paired *t*-test). We speculate that this outcome may reflect RF learning complex patterns that are restricted to one experimental data type (e.g. due to the influence of flanking primer sequences, or the beads used in the selection step), rather than to the RBP.

We note that execution of each of the steps in PRIESSTESS (and each modification tested) involves heuristic choices, and a complete exploration of parameter choices is impractical. The modular nature of the process would, however, facilitate future examination of the components.

### Examination of models and motifs

**Figure 4.**
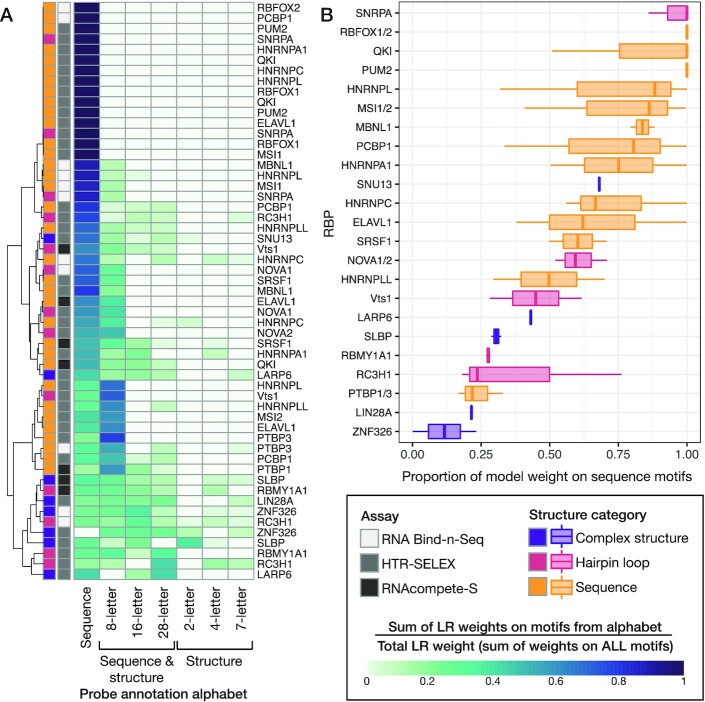
Motif feature weights reveal the secondary structural specificity of RBPs. (**A**) Heatmap displays the proportion of the total feature weights contributed by motifs (PFMs) from each of the seven probe annotation alphabets that are retained in the final PRIESSTESS model. Rows represent the 55 experiments in the benchmarking dataset with the RNA secondary structure category and assay type indicated. The proportion is calculated as the value of the sum of the weights on motifs from a given annotation alphabet divided by the value of the sum of all motif weights. For example, if all motifs retained in the final model are encoded using the sequence alone, the first column will have a value of 1. (**B**) Box plots show the distribution of the proportion of LR model weight on sequence motif features (i.e. left-most heatmap column) across all experiments for each of the RBPs in the benchmarking dataset.

A primary motivation of PRIESSTESS is to facilitate description of the sequence and structure preferences of RBPs. We therefore examined the models in various ways. First, we asked whether the LR weights (i.e., regression coefficients) derived from different RNA sequence/structure alphabets reflected what is known about each of the proteins. Reassuringly, the vocabularies that incorporate RNA structure accounted for a large proportion of the model weight for proteins known to bind specific structures (Figure 4). We next generated a measure of the degree of structural specificity exhibited by an RBP by calculating the sum of the LR model weights on primary sequence motif features retained in the model over the sum of all feature weights in the model (Figure 4B). Surprisingly, this metric often varies across experimental datasets, in particular for ELAVL1, HNRNPL and PCBP1. A possible explanation comes from the fact that the new HTR-SELEX experiments are more likely to display stronger structural specificity for RBPs previously classified as sequence binders (Supplementary Figure S7). We note that the new HTR-SELEX experiments also have lower enrichment in the final cycle, relative to experiments from Jolma *et al.* (11), and are thus less likely to be dominated by a small number of high-affinity sites. The PRIESSTESS models trained on new HTR-SELEX experiments also show the best performance on 8 of the 11 CLIP datasets for sequence binders (Supplementary Figure S7), indicating that they better reflect *in vivo* RNA binding characteristics. Thus, the RNA structure information included in these models may play a greater role than previous *in vitro* data would suggest.

**Figure 5.**
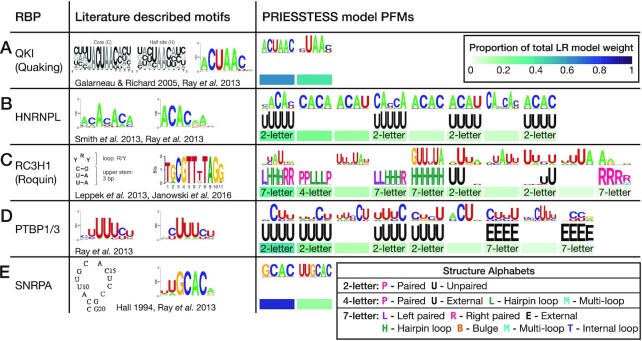
Binding specificities encoded by PRIESSTESS models recapitulate literature knowledge. PRIESSTESS models are compared to literature described binding specificities for (**A**) QKI (9,55), (**B**) HNRNPL (9,52), (**C**) RC3H1 (49,56) and (**D**) PTBP1/3 (9) and (**E**) SNRPA (9,58). Motif features from trained PRIESSTESS models are displayed in decreasing order based on the proportion of the total model weight, which is indicated below each motif. Motif features with zero weights are not shown. For motifs that include structural information, the structural alphabet used in the motif is indicated. As multiple datasets and thus multiple PRIESSTESS models exist for each RBP, the model with the best performance on CLIP data is displayed. As no CLIP data is available for SNRPA, the model with the best performance on the other datasets is displayed. PRIESSTESS models for QKI, HNRNPL, and RC3H1 are trained on data from HTR-SELEX experiments performed for this paper. The PRIESSTESS models for PTBP1/3 and SNRPA are trained on RNAcompete-S data and HTR-SELEX data from Jolma *et al.*, respectively.

To gain a better understanding of the PRIESSTESS models, we manually examined the motifs obtained for a set of proteins with specific characteristics of interest:

**QKI**. QKI, or Quaking, is a well-studied KH domain containing protein named for the tremors seen in mutant mouse models (54). QKI homodimerizes and binds to the bipartite Quaking Response Element (QRE): NACUAAY followed by UAAY 1-20 bases downstream (55). Of the PRIESSTESS models trained on the three QKI RNA-binding datasets, the model that showed the best performance on CLIP data retained exactly two sequence motifs that precisely match the two sites in the QRE (Figure 5A). The PRIESSTESS model performs significantly better than the single most enriched STREME sequence motif (representing the first half of the QRE motif) on both QKI eCLIP datasets (ENCFF786UOW: *P* = 6E−04, ENCFF704OCI: *P* < 1E−10; Delong test).

**HNRNPL**. HNRNPL binds to two CA dinucleotides separated by 0–3 bases (52). PRIESSTESS models trained on *in vitro* datasets retained motifs containing different spacings of CA dinucleotides or single CA dinucleotides (Figure 5B). Comparison of the best performing PRIESSTESS model to the single most enriched STREME sequence motif shows a significant improvement in performance on HNRNPL eCLIP datasets (ENCFF917CBK: *P* < 1E−10, ENCFF266TKW: *P* < 1E−10; Delong test). The ability of the PRIESSTESS models to hold multiple representations of the binding motif presumably allows for better modelling of bipartite binding specificities.

**ROQUIN**. The protein RC3H1, or Roquin, binds the constitutive decay element (CDE) to promote mRNA decay (56). The CDE is a stem-loop structure containing a three-base sequence (YRY) in the hairpin. An alternative and distinct Roquin motif was identified from PAR-CLIP data, a stem–loop containing GUUYUA (49). Examination of the motifs in the Roquin PRIESSTESS model that best predicts the CLIP data reveals motifs that correspond precisely to the CDE and as well as a motif encompassing the alternative stem-loop sequence (GUUNUA) (Figure 5C).

**PTBP1**. PTBP1 has multiple roles in RNA regulation including the repression of alternative exons, stabilization of mRNA, and translation initiation. Each of its four RRM domains binds to a polypyrimidine motif; RRM 1 and 2 are able to bind short motifs within loop structures, whereas RRMs 3 and 4 require longer unstructured polypyrimidine tracts for RNA binding (57). PRIESSTESS models trained on each of the PTBP1 and PTBP3 *in vitro* datasets displayed a strong preference for binding explicitly unpaired polypyrimidine tract sequences (Figure 5D and Supplementary Figure S8). These models showed a significant improvement over models trained using only sequence motifs on eCLIP data, increasing AUROC by up to 0.03. It has previously been reported that many RNA sequence binding proteins prefer to bind to unpaired bases (5), but the reliance on unpaired nucleotides for PTBP1 binding is much stronger than other RBPs in the benchmarking dataset. As RRMs 3 and 4 dimerize, and all four RRMs make contact with the RNA, it may be more difficult to induce a conformational change in the RNA upon binding, thus necessitating that the RNA is intrinsically unpaired.

**SNRPA**. As a component of the U1 snRNP, SNRPA binds to the stem-loop II of the U1 snRNA and is essential for splicing (48,58). It also binds to an internal loop in its own 3’UTR with the same sequence as the U1 snRNA stem-loop. PRIESSTESS models trained on three different *in vitro* SNRPA binding datasets, however, contain little to no structural information. Two models contain only primary sequence motifs which include the known loop sequence, but do not incorporate the stem sequence or any secondary structure information (Figure 5E, Supplementary Figure S8). The third model contains mainly primary nucleotide motifs but does contain two motifs with lower weights that indicate the sequence is unpaired (Supplementary Figure S8). Previous RNAcompete experiments also revealed only a single primary sequence motif which is virtually identical to the motifs identified by PRIESSTESS models (9).

These examples illustrate that PRIESSTESS is capable of modelling both simple and complex RNA-binding specificities, by allowing models to retain only the most important motifs. RNA structure can be omitted for RBPs like QKI and SNRPA, while still maintaining the correct level of structural specificity (paired vs. unpaired or fine-grained structural alphabets) for PTBP1/3 and RC3H1. Importantly, these models also allow for multiple distinct motifs to be modelled when relevant (RC3H1), which can capture components of bipartite motifs as well (QKI, HNRNPL). PRIESSTESS model motifs for all 55 benchmarking dataset experiments are compiled in Supplementary Figure S8 and are available on GitHub (see Availability).

### Models for all published *in vitro* data

**Figure 6.**
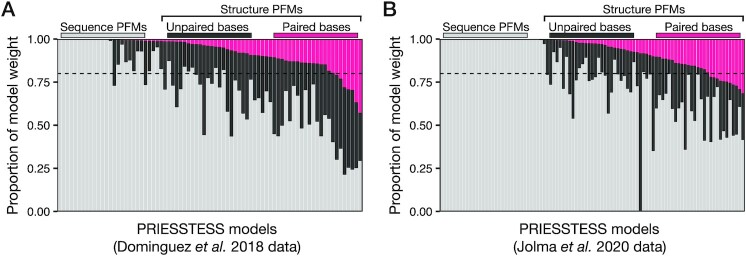
Effect of accessibility on RBP binding propensity. Stacked bar charts display the proportion of PRIESSTESS model weight on sequence PFMs, and on unpaired and paired bases in structure inclusive PFMs, for all published RBNS (**A**) and HTR-SELEX (**B**) datasets. Dashed lines indicate 80% of model weight. Paired bases are ‘P’ in the 2- and 4-letter structural alphabets, and ‘L’ and ‘R’ in the 7-letter structural alphabets. The information content contributed by paired bases is multiplied by the weight placed on the PFM by the model to determine the proportion of model weight on paired bases.

We next applied PRIESSTESS more broadly, examining all the previously published RBNS and HTR-SELEX datasets (7,11). We first asked whether the LR weights on PFMs from the seven sequence/structure alphabets confirm the importance of RNA structure, as previously documented (7,11) (Supplementary Figure S9). Of the RBNS experiments in the upper and lower quartile with respect to enrichment of the top *k*-mer in a paired RNA context, as defined in Dominguez *et al.*, 83% also show structural context sensitivity in the PRIESSTESS model. Similarly, 72% of HTR-SELEX experiments identified as displaying hairpin loop structure specificity in the Jolma *et al.* analysis also displayed structural sensitivity in the corresponding PRIESSTESS model. Overall, models for 83% of RBNS datasets and 68% of HTR-SELEX datasets contain at least one motif with secondary structure information (Figure 6), indicating that RNA secondary structure plays some role in proteins binding to RNA, for the majority of RBPs in each dataset.

We also examined the specific RNA structure attributes contained in the models. To investigate whether the structural information retained in the PRIESSTESS models mainly describes accessibility, which is known to impact RNA binding propensity of RBPs (5), we calculated the proportion of the model weight on unpaired bases (nucleotides in external or loop regions) vs. paired bases in structure inclusive motifs. The vast majority (90%) of the models placed over 80% of the total model weight on sequence motifs and unpaired bases in structure inclusive motifs, indicating that the studied RBPs are predominantly recognizing accessible primary nucleotide sequence (Figure 6). The 18 RBP models that placed over 20% of the model weight on paired bases were consistent with known binding specificities. These RBPs included the dsRNA binder ZNF326 (7); LARP6, which binds an internal loop within a stem structure (4); and RBMY1E, a close paralogue of hairpin loop binding protein RBMY1A1 (59). Thus, while PRIESSTESS can identify RBP structural preferences, the data examined is dominated by a preference for unpaired bases.

An intriguing outcome of the analysis is that the number of retained motif features in PRIESSTESS models is usually much greater than one (median = 7). HTR-SELEX experiment models retained a median of 6 PFMs, whereas RBNS experiments retained a median of 8 PFMs (Supplementary Figure S10A). As some of these motifs may have small contributions to the model, we asked how many motifs account for 90% of the total LR model weight, which dropped the median retained PFMs to 3 for models derived from both HTR-SELEX and RBNS datasets. Only 11% of all models have a single PFM that accounts for over 90% of the model weight (Supplementary Figure S10B). The fact that a single motif is rarely selected—and that PRIESSTESS performs almost universally better than BEESEM, which was designed to produce a single optimal motif – indicates that complexities in RNA–protein interaction are rarely represented well by a single PFM.

## DISCUSSION

PRIESSTESS represents the first systematic method that determines the appropriate number of motifs and level of structural inclusion for individual RBP models without human intervention. The models generated by PRIESSTESS have good predictive ability and are interpretable, as demonstrated using a new curated benchmarking dataset consisting of multiple datatypes for a panel of diverse RBPs that vary in RNA binding modes (structure specificity, bipartite specificity, etc.). This dataset represents an important resource that can be used to assess and compare computational methods and assays. PRIESSTESS also produces a measure of secondary structure contribution to RBP binding and can concurrently model multiple distinct motifs for a single RBP, facilitating modelling of complex binding specificities. The modularity of PRIESSTESS allows for alteration of various aspects of the pipeline, e.g. the method used for RNA folding or for the identification of enriched motifs. Importantly, as PRIESSTESS was not designed for a specific type of RBP-RNA binding assay, it is amenable to any experimental data that can be represented as RBP-bound and background sets of sequences.

PRIESSTESS models reveal the importance of secondary structure to the intrinsic binding specificities of a large proportion of RBPs. Most of the secondary structure motifs, however, represent preference for unpaired primary nucleotide sequence, consistent with prior observations that RBPs generally prefer unpaired sites *in vivo* (5). Discounting those cases where the RBP binds to unpaired primary sequence, only 10% have an apparent requirement for binding to an RNA structure or structural context. This outcome would not have been readily apparent unless the model produced a small number of human readable motifs.

PRIESSTESS represents an intermediate between single motifs and full *k*-mer based models. As such, it does not capture the full granularity and detail that may be achieved by methods such as RCK, GraphProt, and pysster, although it performs similarly in cross-platform comparisons. Additionally, PRIESSTESS is uniformly superior to BEESEM in our analyses, indicating that, as with transcription factors (60,61), inclusion of diverse binding modes is important in representing RNA binding activity. We note that a different number of motifs is typically obtained by PRIESSTESS for the same protein analyzed on different platforms, or the same method by different labs. We attribute this outcome to the fact that PRIESSTESS is dependent on the initial motif finding stage, which will inevitably produce subtle variation in the motifs due to experimental variation, leading to differences in the LR outcome. Stabilization of the retained motifs across datasets may represent a future goal. Additional challenges include further addressing the ensemble of structures, adapting the method to datasets with continuous affinity values, and including tertiary structure annotations, which has been shown to improve site recognition for some RBPs (62).

## DATA AVAILABILITY

PRIESSTESS is available in the GitHub repository: https://github.com/kaitlin309/PRIESSTESS.

## ACCESSION NUMBERS

Data from HTR-SELEX performed for this paper are deposited on the European Nucleotide Archive under the accession PRJEB47428.

## Supplementary Material

gkac694_Supplemental_Files
