# Supplementary material for: PRIESSTESS: interpretable, high-performing models of the sequence and structure preferences of RNA-binding proteins
Source: Nucleic Acids Res. 2022 Aug 26;50(19):e111. doi: 10.1093/nar/gkac694 (PMC9638913; doi:10.1093/nar/gkac694)
Supplement: gkac694_Supplemental_Files [file gkac694_supplemental_files.zip › PRIESSTESS.Supplementary.Figures.pdf]

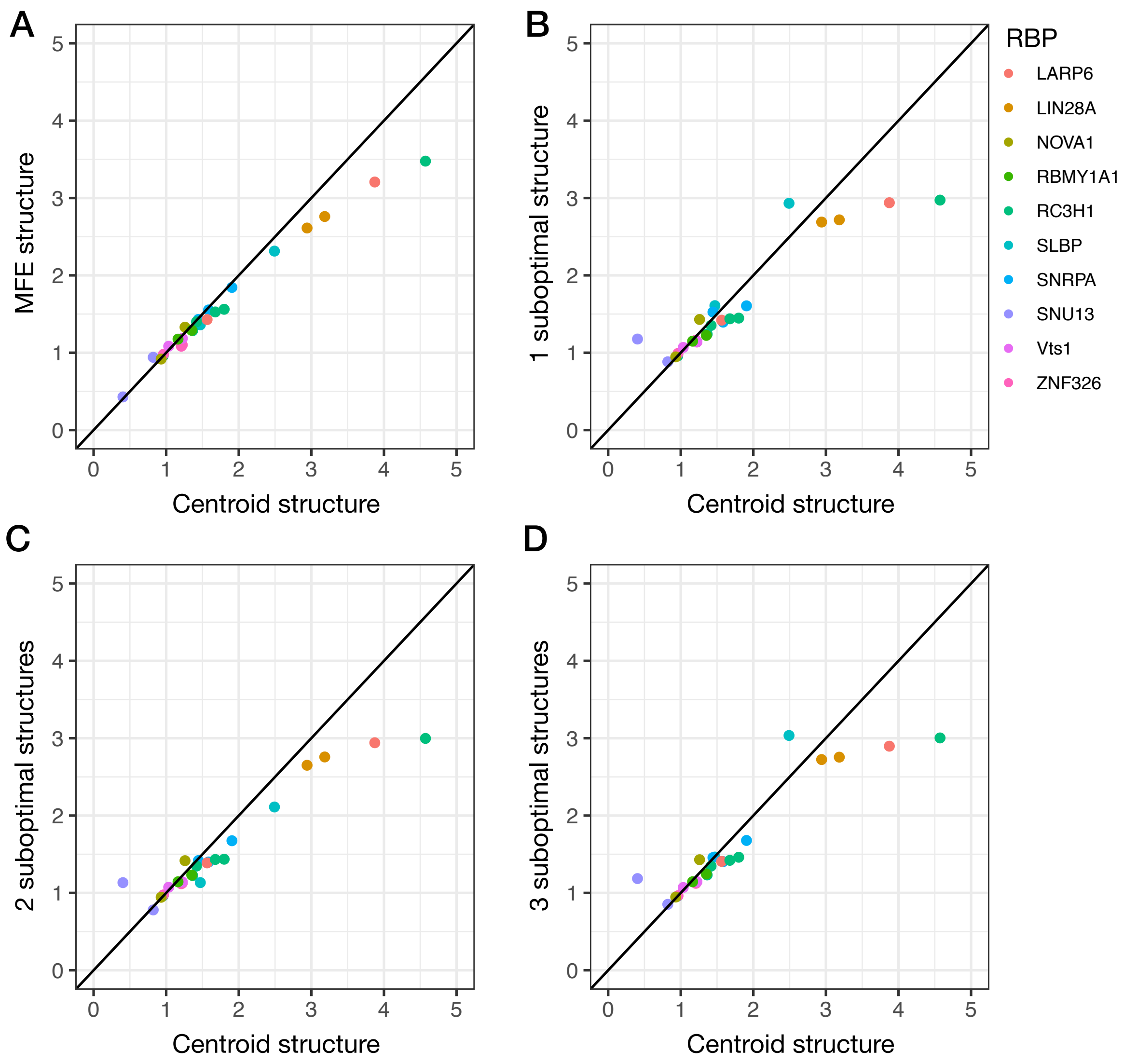

A
B
C
D
MFE structure
1 suboptimal structure
2 suboptimal structures
3 suboptimal structures
Centroid structure
0
1
2
3
4
5
RBP
LARP6
LIN28A
NOVA1
RBMY1A1
RC3H1
SLBP
SNRPA
SNU13
Vts1
ZNF326

Figure S1. Centroid structure best represents complex secondary structure specificities. Comparison of sequence-structure motif enrichment under different representations of secondary structure. Regular expressions representing the literature-described motifs for the 10 hairpin loop or complex structure binding RBPs in the benchmarking dataset were counted in the positive and negative sets to determine fold-change. Fold-change is compared between the centroid structure and the MFE structure (**A**), or 1, 2 or 3 randomly sampled suboptimal structures (**B, C, D**).

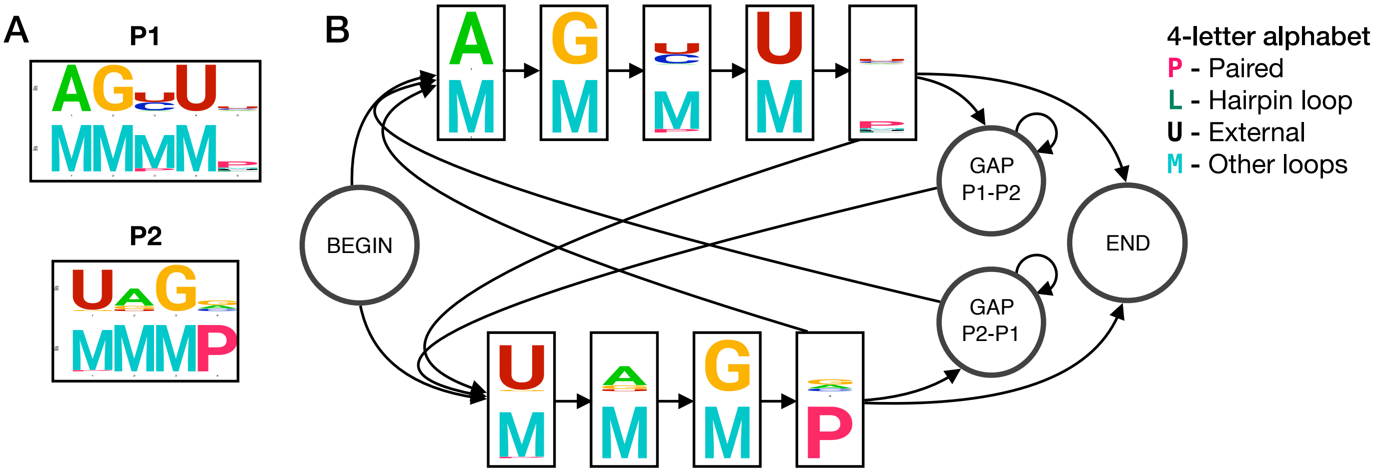

A
P1
P2
B
BEGIN
GAP P1-P2
GAP P2-P1
END
4-letter alphabet
P - Paired
L - Hairpin loop
U - External
M - Other loops

Figure S2. HMM architecture. Each of the 7 HMMs is initialized using up to 10 enriched PFMs. In this figure, the HMM architecture is demonstrated using only two PFMs, P1 & P2 (left). The HMM is initialized as depicted on the left. States in the model include recursive "begin" and "end" states, a state for each position in the PFMs, and recursive "switch" states. Non-zero transition probabilities between states are depicted by arrows. Recursive gap states exist for each pair of PFMs in the model and allow modelling of gaps between two PFMs. The observations for the HMM are the letters in the alphabet from which we identified the PFMs. Emission probabilities in PFM states are equal to the probabilities in the PFM, and for all other states the probabilities for each observation are set to be 1/(alphabet length).

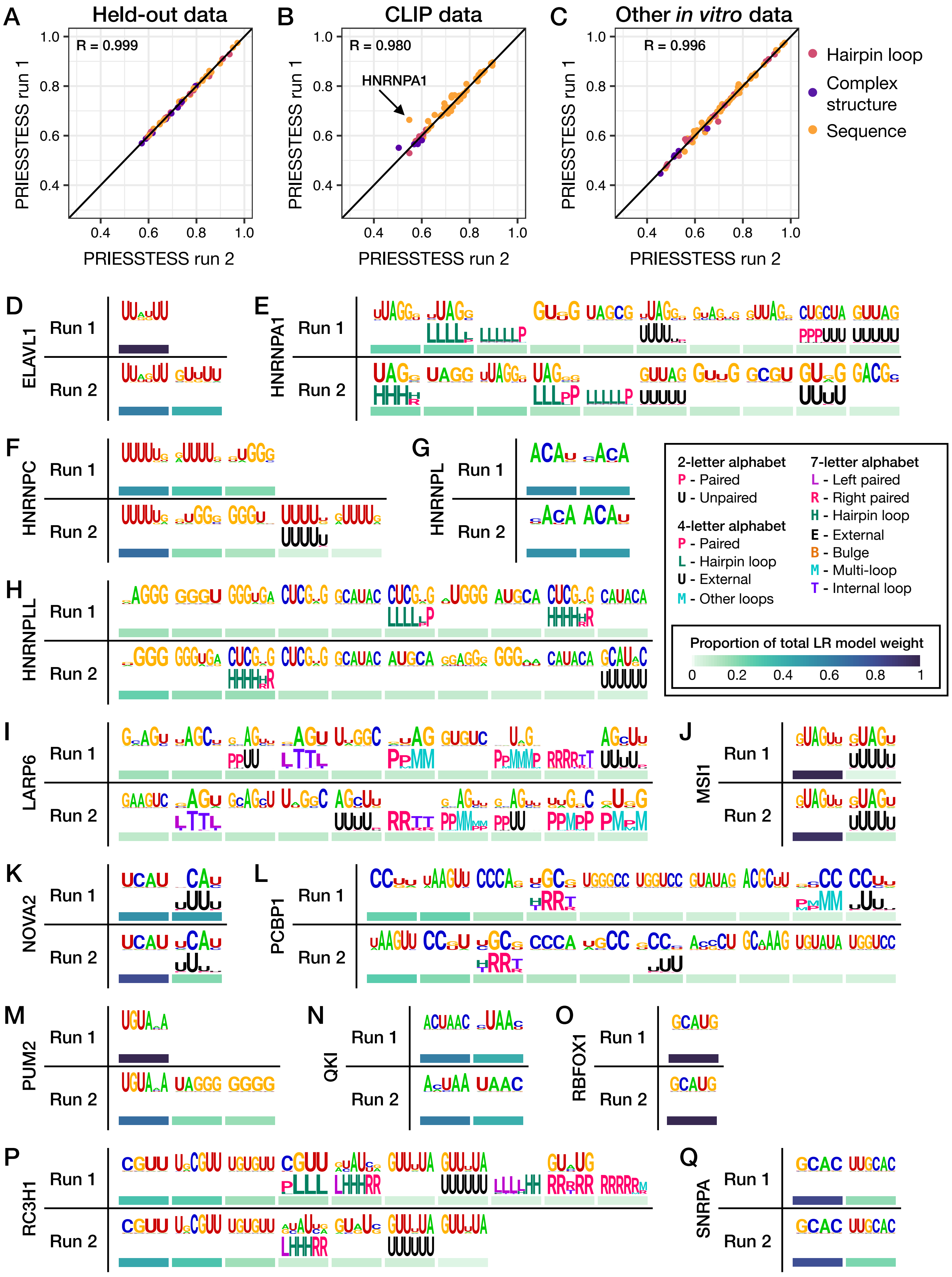
A
Held-out data
R = 0.999
PRIESSTESS run 1
PRIESSTESS run 2
B
CLIP data
R = 0.980
HNRNPA1
PRIESSTESS run 1
PRIESSTESS run 2
C
Other in vitro data
R = 0.996
PRIESSTESS run 1
PRIESSTESS run 2
Hairpin loop
Complex structure
Sequence
D
ELAVL1
E
HNRNPA1
F
HNRNPC
G
HNRNPL
H
HNRNPLL
I
LARP6
J
MSI1
K
NOVA2
L
PCBP1
M
PUM2
N
QKI
O
RBFOX1
P
RC3H1
Q
SNRPA
Run 1
Run 2
2-letter alphabet
P - Paired
U - Unpaired
4-letter alphabet
P - Paired
L - Hairpin loop
U - External
M - Other loops
7-letter alphabet
L - Left paired
R - Right paired
H - Hairpin loop
E - External
B - Bulge
M - Multi-loop
T - Internal loop
Proportion of total LR model weight
0 0.2 0.4 0.6 0.8 1

Figure S3. Reproducibility of PRIESSTESS models with different train-test splits. Each of the 55 benchmarking experiments was randomly split into train and test sets twice and a PRIESSTESS model was trained for each split (denoted run 1 and run 2). Performance of each pair of PRIESSTESS models is compared via AUROC on **A**, held-out data, **B**, CLIP data, **C**, data from other *in vitro* experiments. Pearson correlation is displayed on each plot. The pair of PRIESSTESS models with the greatest difference in performance on CLIP data is pointed out in **B** (HNRNPA1, Jolma *et al.* 2020 experiment). Pairs of PRIESSTESS models generated from RNAcompete-S experiments, RNA Bind-n-Seq experiments, and the 23 HTR-SELEX experiments performed for this paper have a mean difference of 0.009 AUROC on CLIP data. Pairs of models generated with HTR-SELEX data from Jolma *et al.* 2020 have a mean difference of 0.019. **D-Q** compare the motifs in pairs of PRIESSTESS models for each RBP in the Jolma *et al.* 2020 HTR-SELEX dataset (which show the greatest divergence overall). Note that **E** contains the motifs from the pair of models with the greatest divergence in performance on CLIP data.

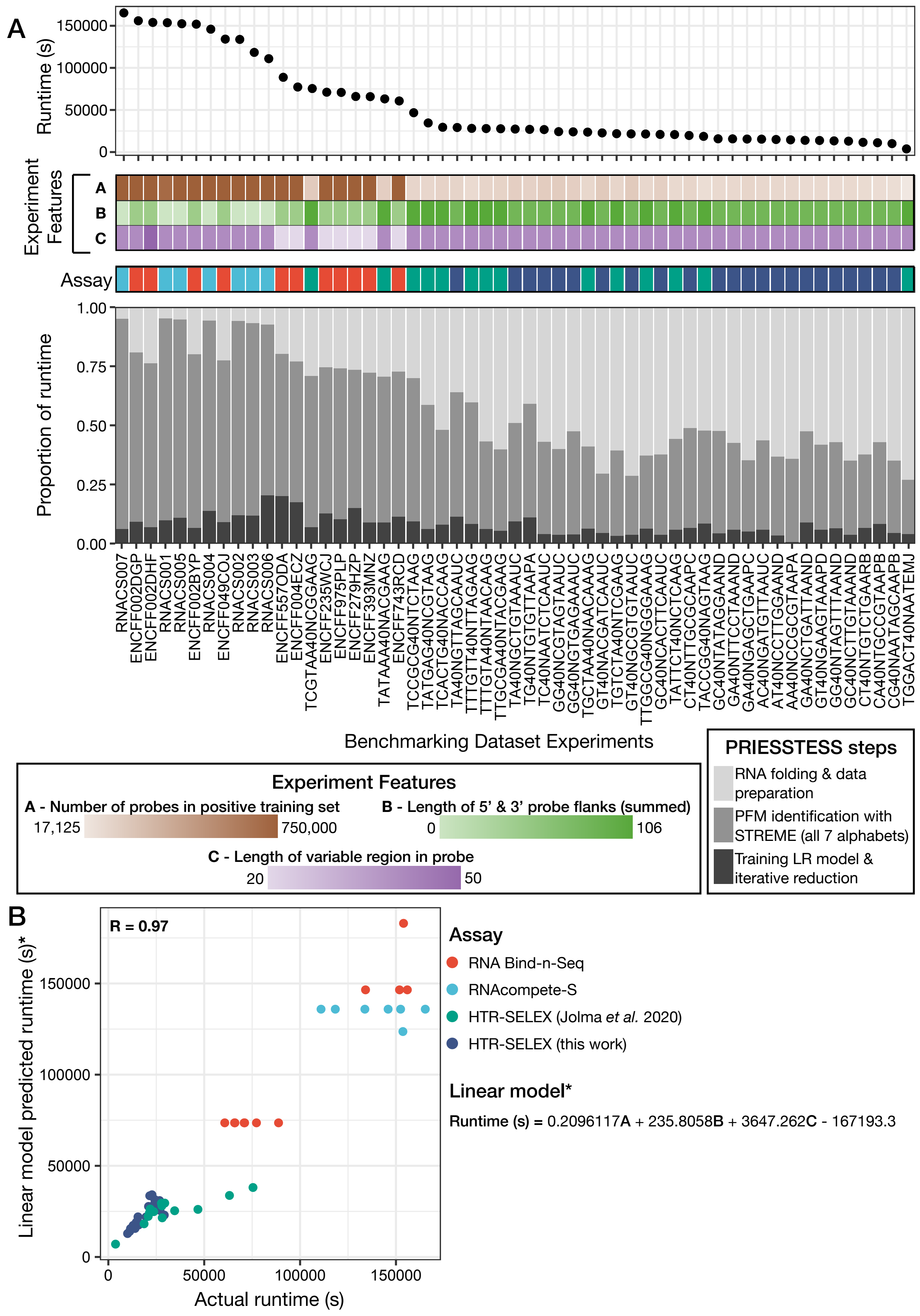
A
Runtime (s)
150000
100000
50000
0
Experiment Features
A
B
C
Assay
Proportion of runtime
1.00
0.75
0.50
0.25
0.00
RNACS007
ENCFF002DGP
ENCFF002DHF
RNACS001
RNACS005
ENCFF002BYP
RNACS004
ENCFF049COJ
RNACS002
RNACS003
RNACS006
ENCFF557ODA
ENCFF004ECZ
TCGTAA40NCGGAAG
ENCFF235WCJ
ENCFF975PLP
ENCFF279HZP
ENCFF393MNZ
TATAAA40NACGAAG
ENCFF743RCD
TCCGCG40NTCTAAG
TATGAG40NCGTAAG
TCACTG40NACCAAG
TA40NGTTAGCAAUC
TTTGTT40NTTAGAAG
TTTGTA40NTAACAAG
TTGCGA40NTACGAAG
TA40NGCTGTAAAUC
TG40NTGTGTTAAPA
TC40NAATCTCAAUC
GG40NCGTAGTAAUC
GG40NGTGAGAAAUC
TGCTAA40NAACAAAG
GT40NACGATCAAUC
TGTCTA40NTCGAAG
GT40NGCGTGTAAUC
TTGGCG40NGGGAAAG
GC40NCACTTCAAUC
TATTCT40NCTCAAG
CT40NTTGCGCAAPC
TACCGG40NAGTAAG
GC40NTATAGGAAND
GA40NTTCCTAAAND
GA40NGAGCTGAAPC
AC40NGATGTTAAUC
AT40NCCTTGGAAND
AA40NCCGCGTAAPA
GA40NCTGATTAAND
GT40NGAAGTAAAPD
GG40NTAGTTTAAND
GC40NCTTGTAAAND
CT40NTGTCTGAARB
CA40NTGCCGTAAPB
CG40NAATAGCAAPB
TGGACT40NAATEMJ
Benchmarking Dataset Experiments
Experiment Features
A - Number of probes in positive training set
17,125
750,000
B - Length of 5' & 3' probe flanks (summed)
0
106
C - Length of variable region in probe
20
50
PRIESSTESS steps
RNA folding & data preparation
PFM identification with STREME (all 7 alphabets)
Training LR model & iterative reduction
B
R = 0.97
Linear model predicted runtime (s)*
150000
100000
50000
0
Actual runtime (s)
0
50000
100000
150000
Assay
RNA Bind-n-Seq
RNAcompete-S
HTR-SELEX (Jolma et al. 2020)
HTR-SELEX (this work)
Linear model*
Runtime (s) = 0.2096117A + 235.8058B + 3647.262C - 167193.3

Figure S4. PRIESSTESS runtime analysis. A, from top to bottom: the runtime of PRIESSTESS for each of the 55 experiments in the benchmarking dataset, experimental features that affect the runtime of PRIESSTESS, the type of assay (see legend in **B**), and the proportion of the runtime spent in each of the three major steps of PRIESSTESS. The experiment features are: (A) the number of probes in the positive training set (this is equal to the number of probes in the negative training set), this feature increases runtime across all steps; (B) the length of 5' and 3' sequences that flank the variable portion of the RNA probes in the experiment, this increases RNA folding runtime; (C) the length of the variable portion of the RNA probes used in the experiment, this feature increases runtime across the RNA folding and PFM identification steps. For each experiment, PRIESSTESS was run on a single Intel Skylake core (2.4GHz). **B**, comparison of PRIESSTESS runtime to predicted runtime from a linear model fitted using the three experiment features (A-C).

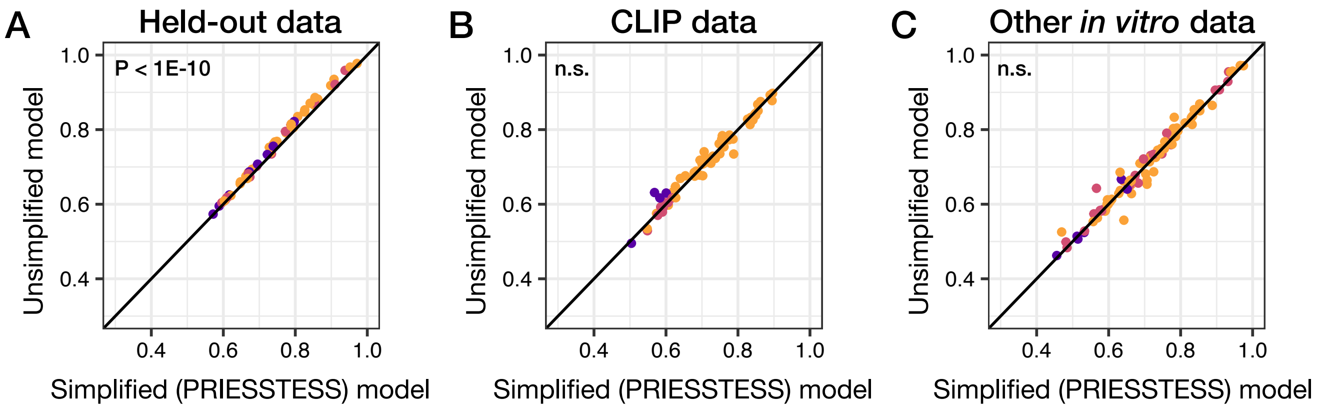
A
Held-out data
P < 1E-10
B
CLIP data
n.s.
C
Other *in vitro* data
n.s.
Unsimplified model
Simplified (PRIESSTESS) model
0.4
0.6
0.8
1.0

Figure S5. Model simplification step does not alter predictive power on other datasets. Comparison of performance (as the AUROC) of simplified PRIESSTESS models to models generated without iterative scaling of L1 strength (unsimplified models) on **A**, held-out data, **B**, CLIP data, **C**, other *in vitro* data. P-values resulting from two-sided paired t-tests are shown on each plot.

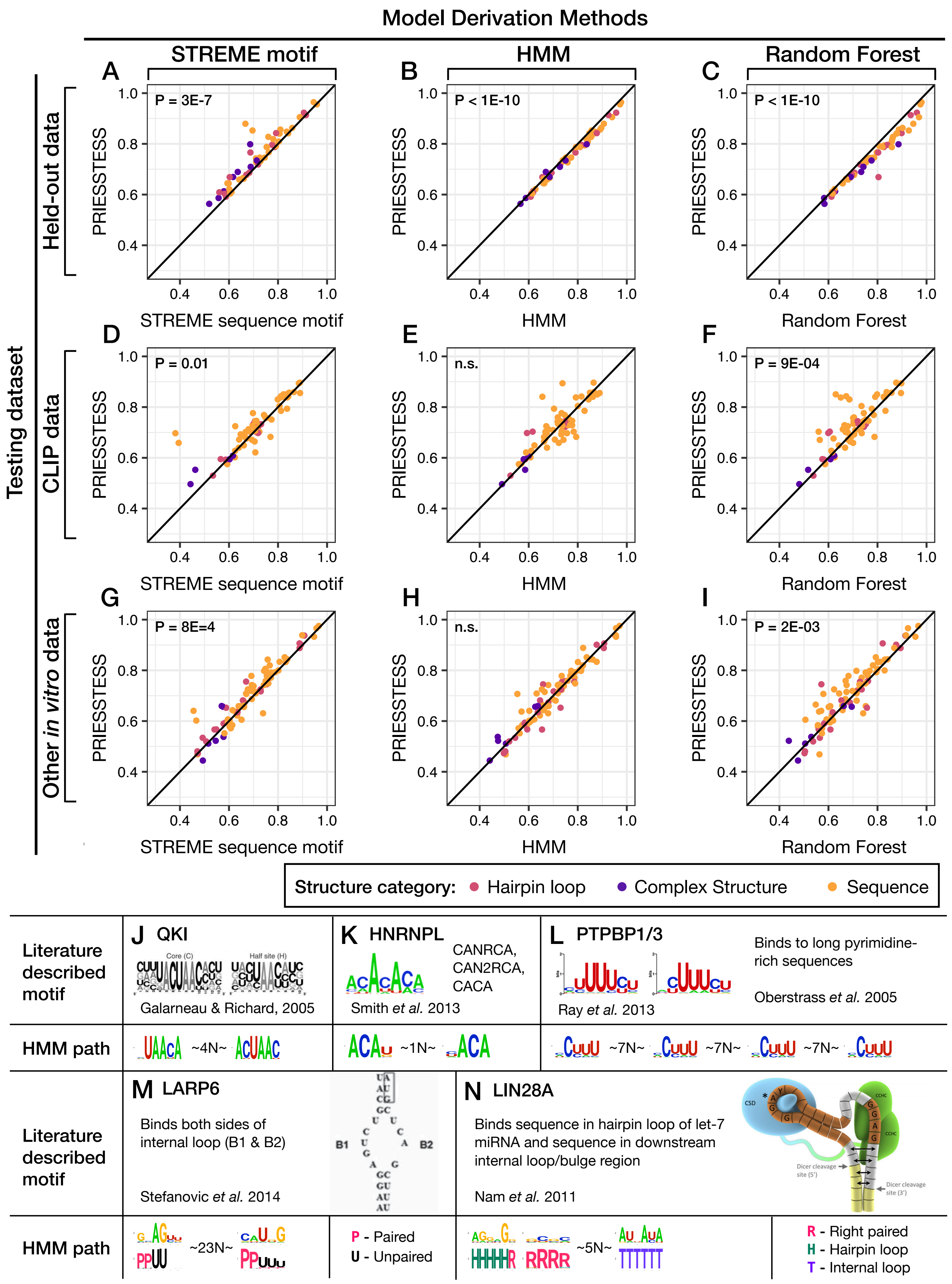
Model Derivation Methods
STREME motif
HMM
Random Forest
A
B
C
Testing dataset
Held-out data
CLIP data
Other in vitro data
PRIESSTESS
P = 3E-7
P < 1E-10
P < 1E-10
STREME sequence motif
HMM
Random Forest
D
E
F
P = 0.01
n.s.
P = 9E-04
G
H
I
P = 8E=4
n.s.
P = 2E-03
0.4
0.6
0.8
1.0
Structure category: Hairpin loop Complex Structure Sequence
Literature described motif
HMM path
J QKI
Core (C)
Half site (H)
Galarneau & Richard, 2005
K HNRNPL
CANRCA, CAN2RCA, CACA
Smith et al. 2013
L PTPBP1/3
Ray et al. 2013
Binds to long pyrimidine-rich sequences
Oberstrass et al. 2005
~4N~
~1N~
~7N~
M LARP6
Binds both sides of internal loop (B1 & B2)
Stefanovic et al. 2014
B1
B2
N LIN28A
Binds sequence in hairpin loop of let-7 miRNA and sequence in downstream internal loop/bulge region
Nam et al. 2011
CSD
CCHC
Dicer cleavage site (5')
Dicer cleavage site (3')
~23N~
P - Paired
U - Unpaired
~5N~
R - Right paired
H - Hairpin loop
T - Internal loop

Figure S6. Dissection of PRIESSTESS. PRIESSTESS models trained on the benchmarking dataset are compared to: the single best sequence motif identified by STREME (**A, D, G**), HMM models (**B, E, H**), and a version of PRIESSTESS in which logistic regression is replaced by random forest trees (**C, F, I**). All models are trained using the same data. Plots show AUROC values of the binding specificity models when applied to three different types of testing data: held-out data from the same experiment as the training data (**A- C**), matching CLIP data (**D-F**), and held-out data from other *in vitro* experiments (same RBP, different assay or different lab) (**G-I**). P-values resulting from two-sided paired t-tests are shown on each plot.
J-N Comparison of top motifs manually extracted from the HMM models trained for QKI, HNRNPL, PTBP1/3, LARP6, and LIN28A, to literature described motifs. Common HMM paths combining 1 or more motifs are shown, with gaps represented by ~#N~ where # is the average number of bases in the gap.

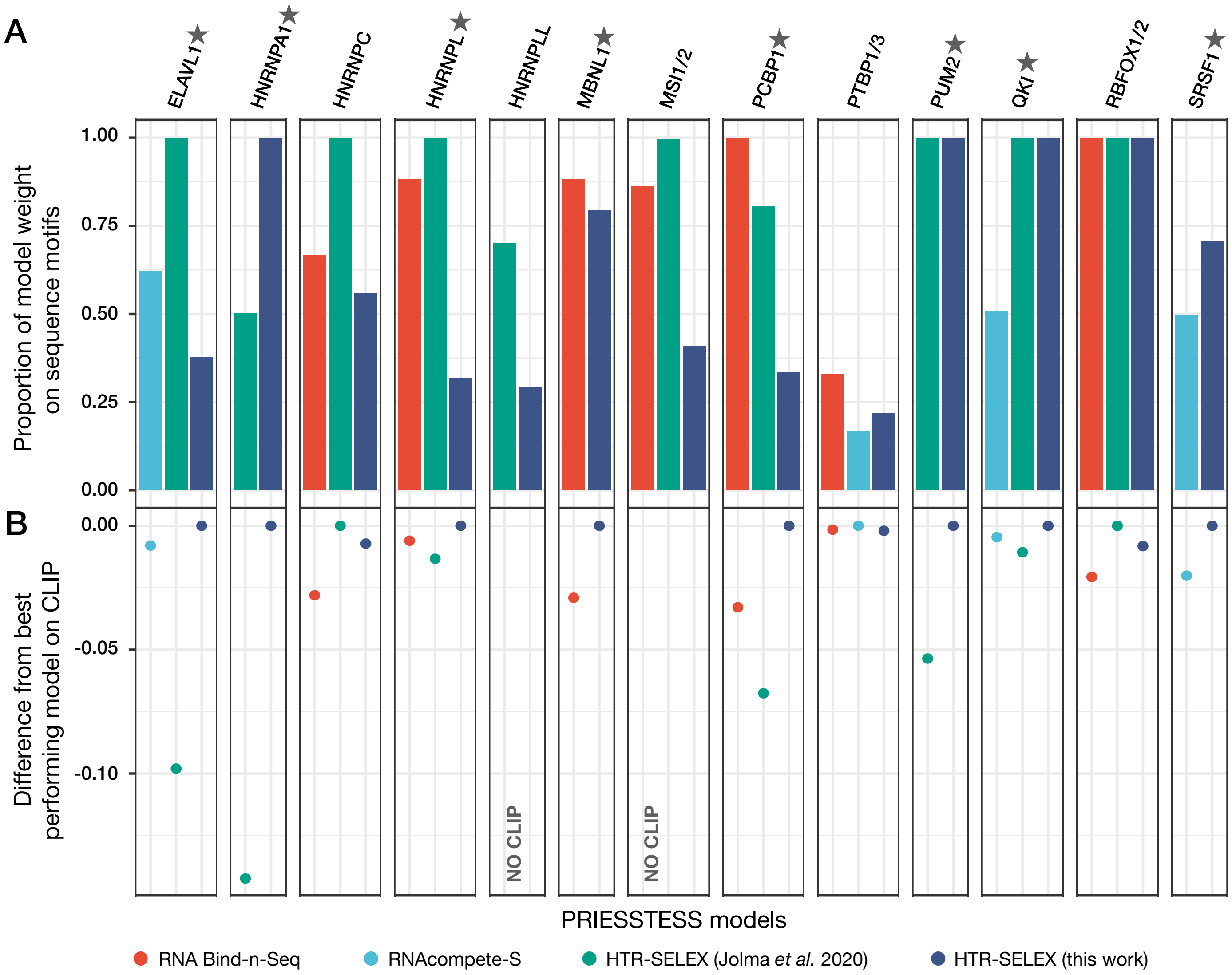
A
B
ELAVL1
HNRNPA1
HNRNPC
HNRNPL
HNRNPLL
MBNL1
MSI1/2
PCBP1
PTBP1/3
PUM2
QKI
RBFOX1/2
SRSF1
Proportion of model weight on sequence motifs
1.00
0.75
0.50
0.25
0.00
Difference from best performing model on CLIP
0.00
-0.05
-0.10
NO CLIP
NO CLIP
PRIESSTESS models
RNA Bind-n-Seq
RNAcompete-S
HTR-SELEX (Jolma et al. 2020)
HTR-SELEX (this work)

Figure S7. Sequence specific RBP assay comparison. A, the degree of structural specificity exhibited by an RBP was calculated by taking the sum of the LR model weights on primary sequence motif features retained in the model over the sum of all feature weights in the model. This proportion of model weight on sequence motifs is compared across PRIESSTESS models for the same RBP with data from different assays. **B**, the difference from the best performing PRIESSTESS model on CLIP per RBP. RBPs for which the PRIESSTESS models trained on new HTR-SELEX experiments shows the best performance are marked with a star.

RBP	Assay
ELAVL1	RNAcompete-S (Cook *et al.* 2017)
ELAVL1	HTR-SELEX (Jolma *et al.* 2020)
**ELAVL1**	HTR-SELEX (this paper)
HNRNPA1	HTR-SELEX (Jolma *et al.* 2020)
**HNRNPA1**	HTR-SELEX (this paper)
HNRNPC	RNA Bind-n-Seq (Dominguez *et al.* 2018)
**HNRNPC**	HTR-SELEX (Jolma *et al.* 2020)
HNRNPC	HTR-SELEX (this paper)
HNRNPL	RNA Bind-n-Seq (Dominguez *et al.* 2018)
HNRNPL	HTR-SELEX (Jolma *et al.* 2020)
**HNRNPL**	HTR-SELEX (this paper)
**HNRNPLL**	HTR-SELEX (Jolma *et al.* 2020)
HNRNPLL	HTR-SELEX (this paper)
**LARP6**	HTR-SELEX (Jolma *et al.* 2020)
LARP6	HTR-SELEX (this paper)
LIN28A	HTR-SELEX (this paper)

PRIESSTESS model PWMs

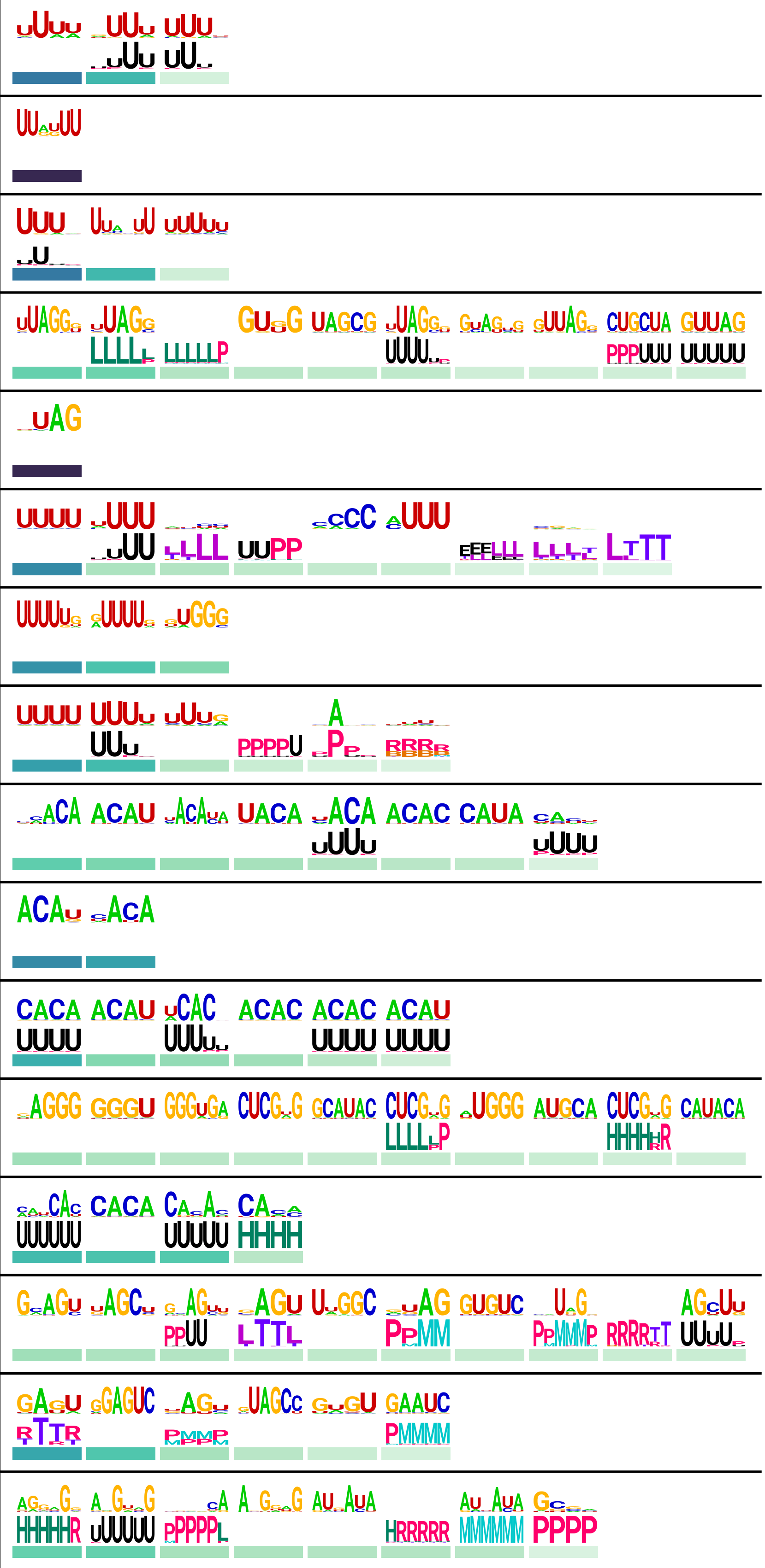

Structure category: Hairpin loop, Complex Structure, Sequence

Proportion of total LR model weight: 0, 0.2, 0.4, 0.6, 0.8, 1

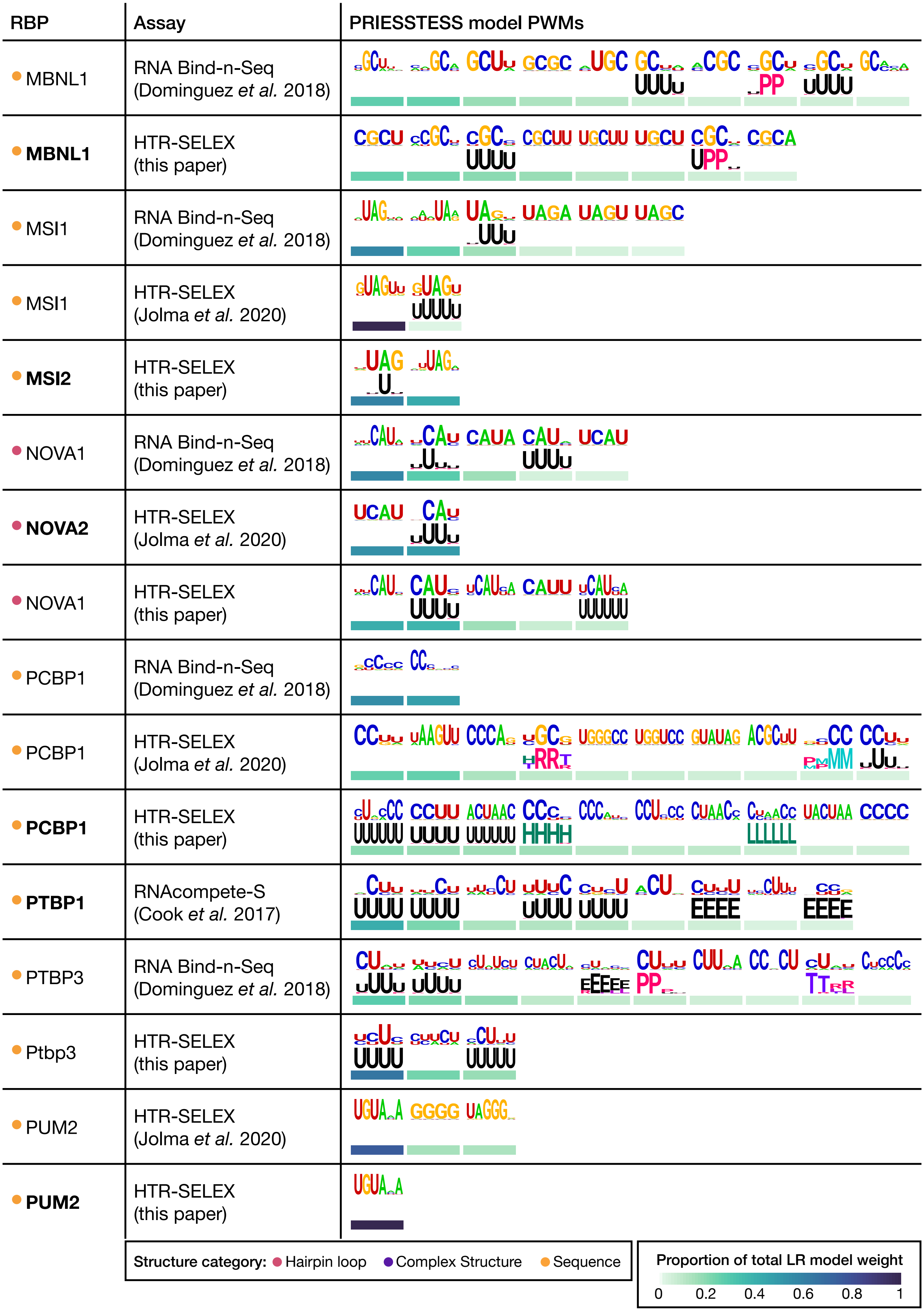
RBP
Assay
PRIESSTESS model PWMs
MBNL1
RNA Bind-n-Seq (Dominguez et al. 2018)
MBNL1
HTR-SELEX (this paper)
MSI1
RNA Bind-n-Seq (Dominguez et al. 2018)
MSI1
HTR-SELEX (Jolma et al. 2020)
MSI2
HTR-SELEX (this paper)
NOVA1
RNA Bind-n-Seq (Dominguez et al. 2018)
NOVA2
HTR-SELEX (Jolma et al. 2020)
NOVA1
HTR-SELEX (this paper)
PCBP1
RNA Bind-n-Seq (Dominguez et al. 2018)
PCBP1
HTR-SELEX (Jolma et al. 2020)
PCBP1
HTR-SELEX (this paper)
PTBP1
RNAcompete-S (Cook et al. 2017)
PTBP3
RNA Bind-n-Seq (Dominguez et al. 2018)
Ptbp3
HTR-SELEX (this paper)
PUM2
HTR-SELEX (Jolma et al. 2020)
PUM2
HTR-SELEX (this paper)
Structure category: Hairpin loop Complex Structure Sequence
Proportion of total LR model weight
0 0.2 0.4 0.6 0.8 1

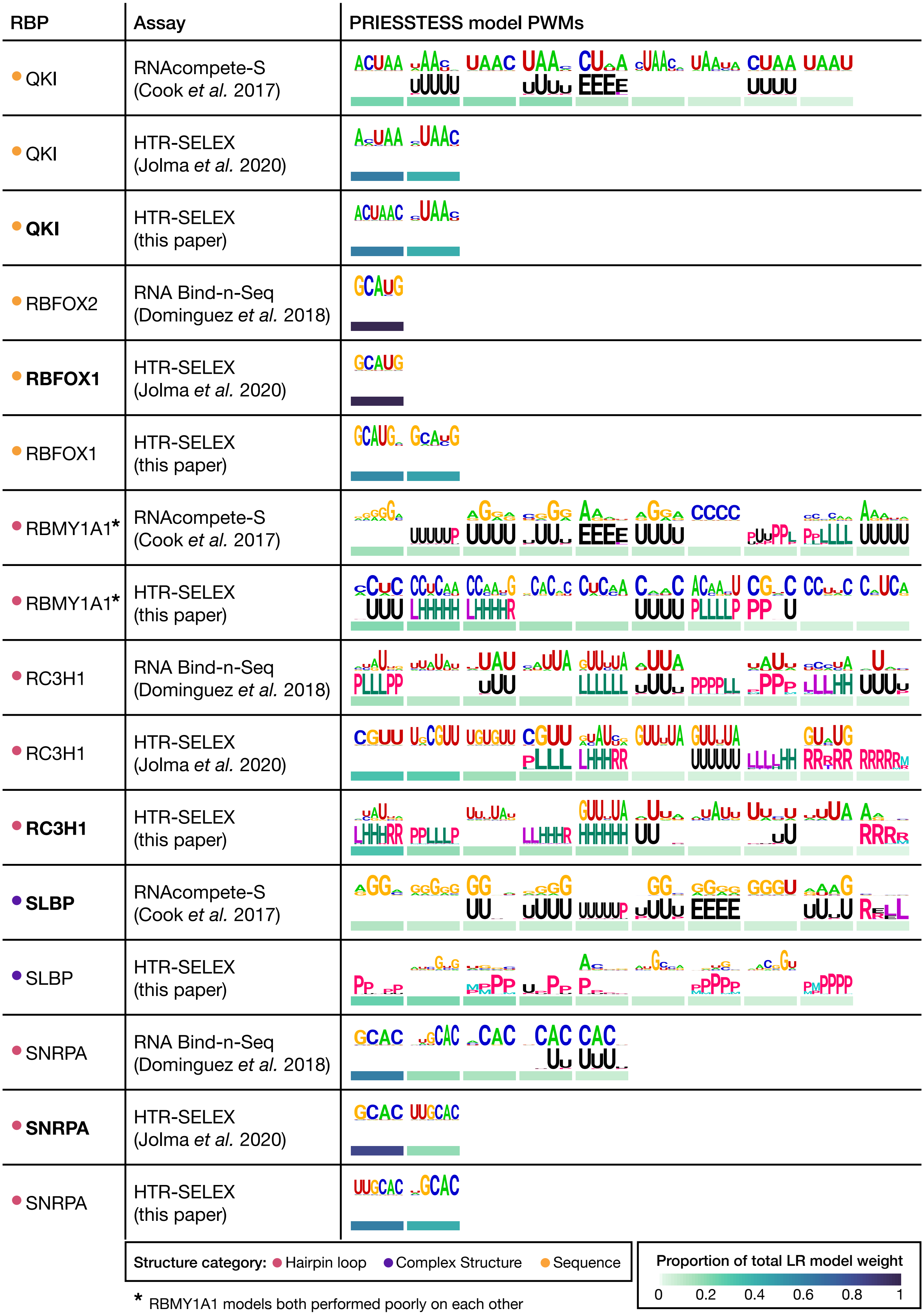
RBP
Assay
PRIESSTESS model PWMs
QKI
RNAcompete-S (Cook et al. 2017)
QKI
HTR-SELEX (Jolma et al. 2020)
QKI
HTR-SELEX (this paper)
RBFOX2
RNA Bind-n-Seq (Dominguez et al. 2018)
RBFOX1
HTR-SELEX (Jolma et al. 2020)
RBFOX1
HTR-SELEX (this paper)
RBMY1A1*
RNAcompete-S (Cook et al. 2017)
RBMY1A1*
HTR-SELEX (this paper)
RC3H1
RNA Bind-n-Seq (Dominguez et al. 2018)
RC3H1
HTR-SELEX (Jolma et al. 2020)
RC3H1
HTR-SELEX (this paper)
SLBP
RNAcompete-S (Cook et al. 2017)
SLBP
HTR-SELEX (this paper)
SNRPA
RNA Bind-n-Seq (Dominguez et al. 2018)
SNRPA
HTR-SELEX (Jolma et al. 2020)
SNRPA
HTR-SELEX (this paper)
Structure category: Hairpin loop Complex Structure Sequence
Proportion of total LR model weight
0 0.2 0.4 0.6 0.8 1
* RBMY1A1 models both performed poorly on each other

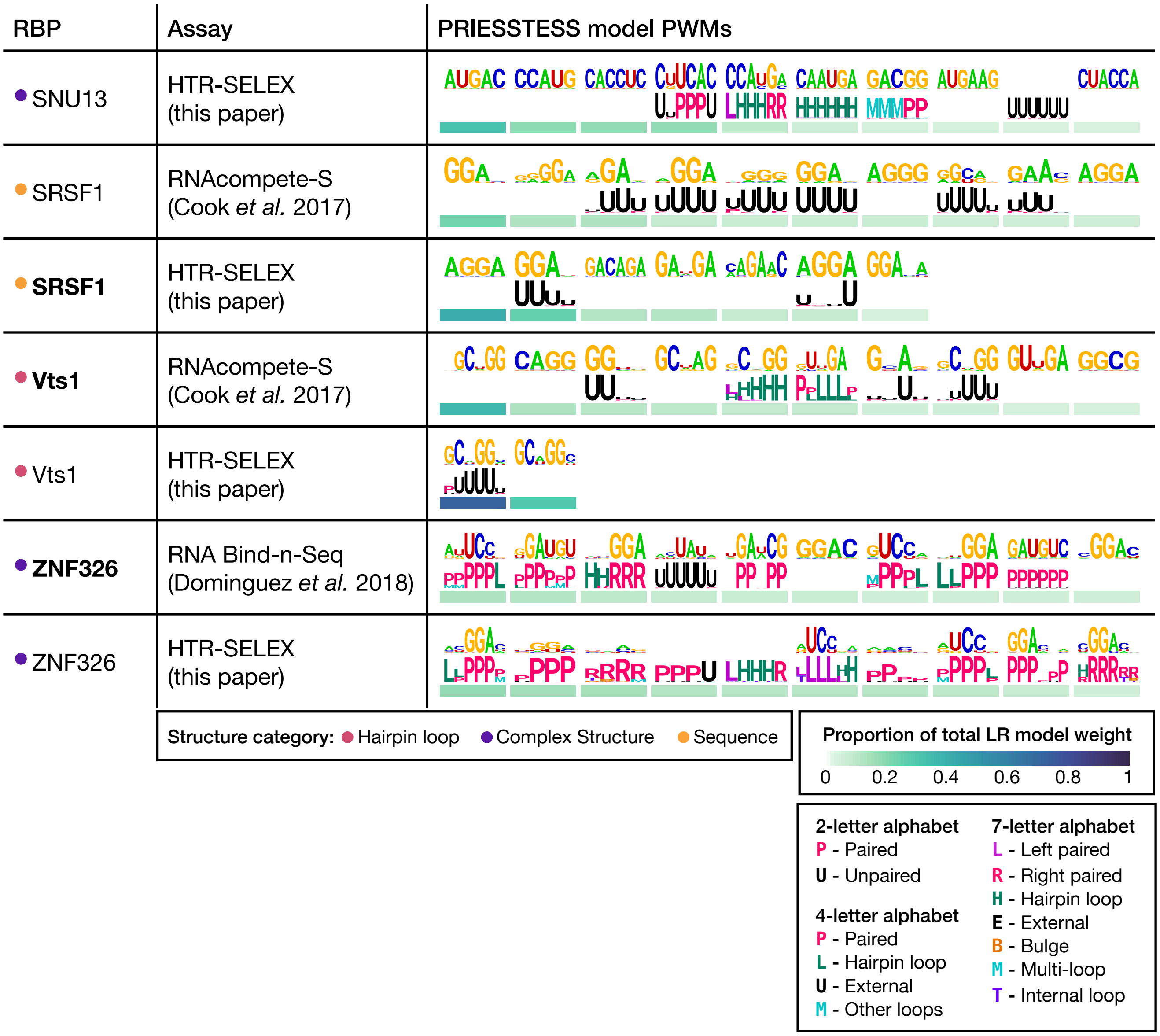

RBP
Assay
PRIESSTESS model PWMs
SNU13
HTR-SELEX (this paper)
SRSF1
RNAcompete-S (Cook et al. 2017)
SRSF1
HTR-SELEX (this paper)
Vts1
RNAcompete-S (Cook et al. 2017)
Vts1
HTR-SELEX (this paper)
ZNF326
RNA Bind-n-Seq (Dominguez et al. 2018)
ZNF326
HTR-SELEX (this paper)
Structure category: Hairpin loop Complex Structure Sequence
Proportion of total LR model weight
0 0.2 0.4 0.6 0.8 1
2-letter alphabet
P - Paired
U - Unpaired
4-letter alphabet
P - Paired
L - Hairpin loop
U - External
M - Other loops
7-letter alphabet
L - Left paired
R - Right paired
H - Hairpin loop
E - External
B - Bulge
M - Multi-loop
T - Internal loop

Figure S8. Binding specificities encoded by PRIESSTESS models for benchmarking dataset experiments. PRIESSTESS models for all RNA Bind-n-Seq, RNAcompete-S, and HTR-SELEX experiments in the benchmarking dataset. Motif features from trained PRIESSTESS models are displayed in decreasing order based on the proportion of the total model weight, which is indicated below each motif. Motif features with zero weights are not shown, and if more than 10 motif features are retained, only the top 10 are displayed. The binding type based on literature review is indicated by a coloured circle to the left of the RBP name. For each RBP with datasets from multiple assays, the dataset which produced the best performing PRIESSTESS model on CLIP data, or if unavailable, on other *in vitro* data, is shown in bold.

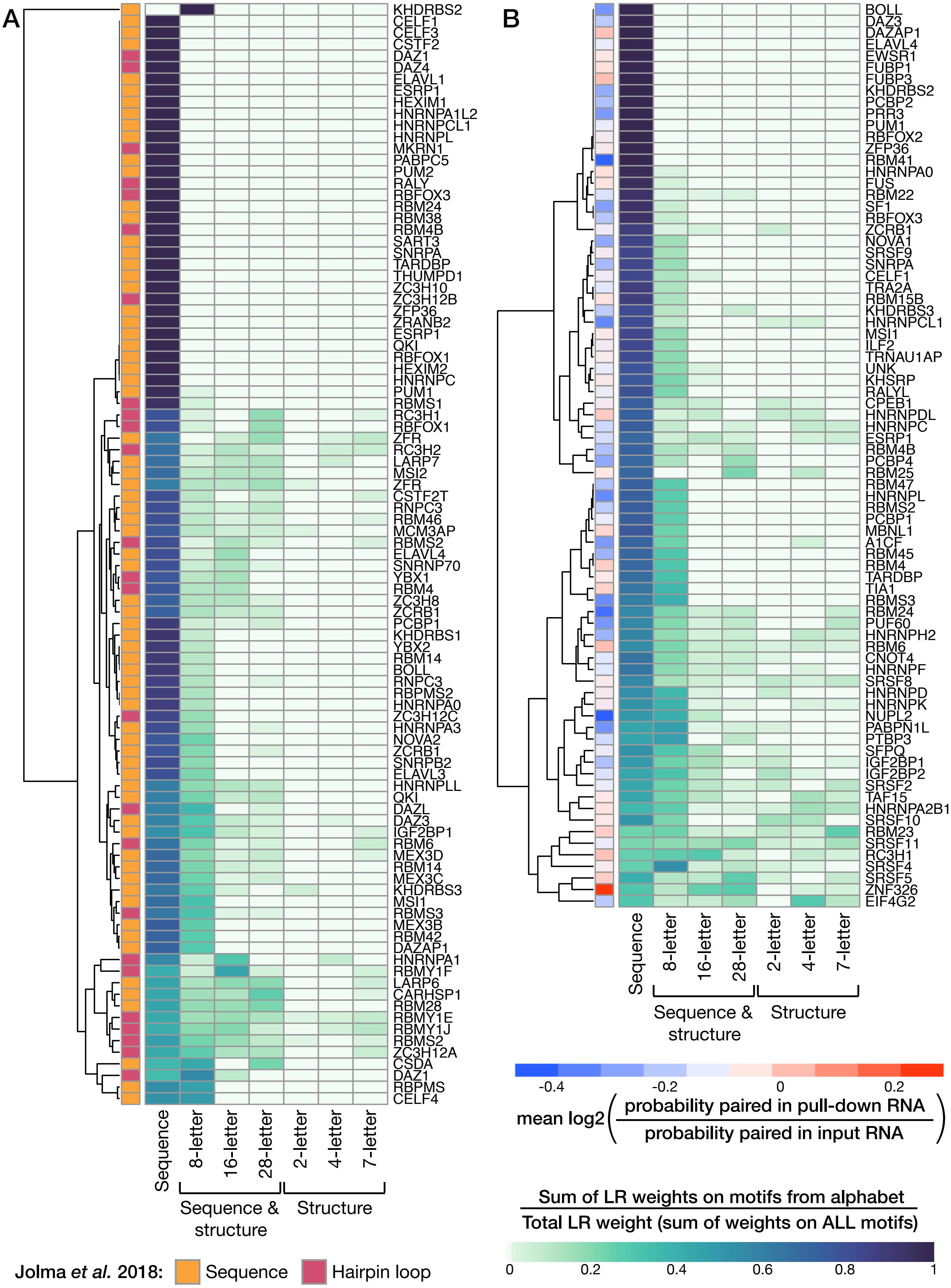

A
KHDRBS2
CELF1
CELF3
CSTF2
DAZ1
DAZ4
ELAVL1
ESRP1
HEXIM1
HNRNPA1L2
HNRNPCL1
HNRNPL
MKRN1
PABPC5
PUM2
RALY
RBFOX3
RBM24
RBM38
RBM4B
SART3
SNRPA
TARDBP
THUMPD1
ZC3H10
ZC3H12B
ZFP36
ZRANB2
ESRP1
QKI
RBFOX1
HEXIM2
HNRNPC
PUM1
RBMS1
RC3H1
RBFOX1
ZFR
RC3H2
LARP7
MSI2
ZFR
CSTF2T
RNPC3
RBM46
MCM3AP
RBMS2
ELAVL4
SNRNP70
YBX1
RBM4
ZC3H8
ZCRB1
PCBP1
KHDRBS1
YBX2
RBM14
BOLL
RNPC3
RBPMS2
HNRNPA0
ZC3H12C
HNRNPA3
NOVA2
ZCRB1
SNRPB2
ELAVL3
HNRNPLL
QKI
DAZL
DAZ3
IGF2BP1
RBM6
MEX3D
RBM14
MEX3C
KHDRBS3
MSI1
RBMS3
MEX3B
RBM42
DAZAP1
HNRNPA1
RBMY1F
LARP6
CARHSP1
RBM28
RBMY1E
RBMY1J
RBMS2
ZC3H12A
CSDA
DAZ1
RBPMS
CELF4
Sequence
8-letter
16-letter
28-letter
2-letter
4-letter
7-letter
Sequence & structure
Structure
Jolma et al. 2018:
Sequence
Hairpin loop
B
BOLL
DAZ3
DAZAP1
ELAVL4
EWSR1
FUBP1
FUBP3
KHDRBS2
PCBP2
PRR3
PUM1
RBFOX2
ZFP36
RBM41
HNRNPA0
FUS
RBM22
SF1
RBFOX3
ZCRB1
NOVA1
SRSF9
SNRPA
CELF1
TRA2A
RBM15B
KHDRBS3
HNRNPCL1
MSI1
ILF2
TRNAU1AP
UNK
KHSRP
RALYL
CPEB1
HNRNPDL
HNRNPC
ESRP1
RBM4B
PCBP4
RBM25
RBM47
HNRNPL
RBMS2
PCBP1
MBNL1
A1CF
RBM45
RBM4
TARDBP
TIA1
RBMS3
RBM24
PUF60
HNRNPH2
RBM6
CNOT4
HNRNPF
SRSF8
HNRNPD
HNRNPK
NUPL2
PABPN1L
PTBP3
SFPQ
IGF2BP1
IGF2BP2
SRSF2
TAF15
HNRNPA2B1
SRSF10
RBM23
SRSF11
RC3H1
SRSF4
SRSF5
ZNF326
EIF4G2
Sequence
8-letter
16-letter
28-letter
2-letter
4-letter
7-letter
Sequence & structure
Structure
-0.4
-0.2
0
0.2
mean log2 (probability paired in pull-down RNA / probability paired in input RNA)
Sum of LR weights on motifs from alphabet / Total LR weight (sum of weights on ALL motifs)
0
0.2
0.4
0.6
0.8
1

Figure S9. RBP RNA secondary structural specificity in published datasets. Heatmaps display the proportion of the total feature weights contributed by motifs (PWMs) from each of the seven probe annotation alphabets that are retained in the final PRIESSTESS model. Rows represent individual experiments. The proportion is calculated as the absolute value of the sum of the weights on motifs from a given annotation alphabet divided by the absolute value of the sum of all motif weights. **A,** Heatmap for all 95 HTR-SELEX experiments (86 unique RBPs) from Jolma *et al.* 2020. Row annotations indicate whether any motifs derived from the experimental data were identified within a hairpin loop in Jolma *et al.* 2020. **B,** Heatmap for all 78 RBPs with published RBNS experiments. The experiment defined as having the best concentration in the supplement of Dominguez *et al. 2018* was used. Row annotations show the log2 fold-change of paired bases in the best 6-mer motif over paired bases in the input, averaged across all 6 bases, taken from Dominguez *et al*. 2018.

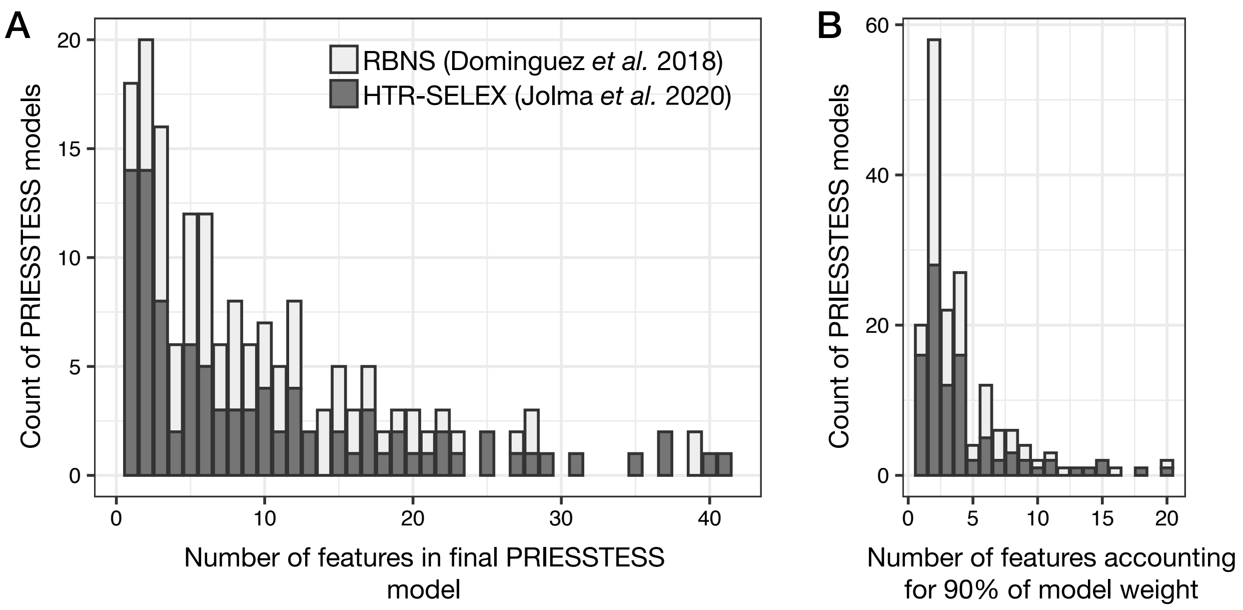
A
B
RBNS (Dominguez et al. 2018)
HTR-SELEX (Jolma et al. 2020)
Count of PRIESSTESS models
Number of features in final PRIESSTESS model
Number of features accounting for 90% of model weight
0
5
10
15
20
30
40
60

Figure S10. Number of motif features retained in PRIESSTESS models. A, Number of features (PFM motifs) retained in PRIESSTESS models for all RBNS experiments from Dominguez *et al.* 2018 and all HTR-SELEX experiments from Jolma *et al.* 2020. **B,** The number of features retained in PRIESSTESS models that account for the first 90% of the LR total model weight.
